# Evolution of pollination by frugivorous birds in Neotropical Myrtaceae

**DOI:** 10.7717/peerj.5426

**Published:** 2018-08-27

**Authors:** María Gabriela Nadra, Norberto Pedro Giannini, Juan Manuel Acosta, Lone Aagesen

**Affiliations:** 1 Unidad Ejecutora Lillo (UEL), CONICET-FML, San Miguel de Tucumán, Tucumán, Argentina; 2 Instituto de Botánica Darwinion (IBODA), CONICET-ANCEFN, San Isidro, Buenos Aires, Argentina; 3 Facultad de Ciencias Naturales e IML, Universidad Nacional de Tucumán, San Miguel deTucumán, Tucumán, Argentina

**Keywords:** Myrrhinium, Fleshy petals, Plant-animal interactions, Phylogeny, Acca, Pimenta-group, Myrteae

## Abstract

Bird pollination is relatively common in the tropics, and especially in the Americas. In the predominantly Neotropical tribe Myrteae (Myrtaceae), species of two genera, *Acca* and *Myrrhinium*, offer fleshy, sugary petals to the consumption of birds that otherwise eat fruits, thus pollinating the plants in an unusual plant-animal interaction. The phylogenetic position of these genera has been problematic, and therefore, so was the understanding of the evolution of this interaction. Here we include new sequences of *Myrrhinium atropurpureum* in a comprehensive molecular phylogeny based on a balanced sample of two plastid and two nuclear markers, with the aim of providing the historical framework of pollination by frugivorous birds in Myrteae. We developed 13 flower and inflorescence characters that comprehensively depict the macroscopic morphological components of this interaction. Bayesian and parsimony phylogenies concur in placing both *Acca* and *Myrrhinium* in a clade with *Psidium* species; with *Myrrhinium* sister to *Psidium*. Mapping of morphological characters indicated some degree of convergence (e.g., fleshy petals, purplish display) but also considerable divergence in key characters that point to rather opposing pollination strategies and also different degrees of specialization in *Acca* versus *Myrrhinium*. Pollination by frugivorous birds represents a special case of mutualism that highlights the evolutionary complexities of plant-animal interactions.

## Introduction

A wide array of evidence supports a strong association between specific floral traits and functional groups of pollinators that exert similar selective pressures on key aspects of the plant reproductive biology (Fenster et al., 2004). One well-known suite of floral traits is associated with the bird pollination syndrome—ornithophily, which is present in some 65 flowering plant families (Cronk & Ojeda, 2008). Most cases likely represent parallel origins of ornithophily from bee-pollinated ancestors (Cronk & Ojeda, 2008). This type of pollination is characterised by a passive pollen transport; nectar is the primary reward for pollinating birds (Stiles, 1981; Proctor, Yeo & Lack, 1996; Armbruster, 2011). Specialized ornithophilous flowers in the classical sense (Faegri & Van der Pijl, 1979) are very often red in colour, tubular and/or pendant or brush-like and produce abundant, diluted nectar; characteristically, these flowers lack scent or chemical attractors that are so frequently associated with other animal pollinators, particularly daytime insects (Faegri & Van der Pijl, 1979; Stiles, 1981; Proctor, Yeo & Lack, 1996; Cronk & Ojeda, 2008). Bird pollination is widespread in tropical areas of the World but reaches its highest diversity in the Americas owing to the evolution of hummingbirds (Trochilidae; Proctor, Yeo & Lack, 1996). Hummingbirds are highly diverse (363 species), small-sized, highly efficient, almost exclusively nectar-feeding birds that are capable of hovering flight—the most expensive mode of locomotion (Norberg, 1994). Flower products are also used by functionally less specialized birds (as compared with hummingbirds) that nonetheless depend completely on flowers: perching birds in different families chiefly, but not exclusively, across the World tropics (see Zanata et al., 2017). Perching nectar-feeding birds include both legitimate pollinators and nectar thieves (Proctor, Yeo & Lack, 1996); the most remarkable groups of perching nectarivorous are from the Old World, and are the sunbirds (Passeriformes, Nectariniidae, 132 species) occurring in tropical Africa, SE Asia and Oceania, and the honeyeaters (Passeriformes, Meliphagidae, 175 species), which are found in SE Asia and Oceania (Zanata et al., 2017). Perching nectarivorous birds also belong in other families such as the New World Thraupidae (Diglossinae, Coerebinae), Icteridae (Icterinae, Cacicinae), and Fringillidae (Carduelinae), among other (see Faegri & Van der Pijl, 1979; Proctor, Yeo & Lack, 1996). With different exploitation strategies, both groups of birds (hovering and perching) visit flowers of different morphologies and inflorescences that also present diverse architectures (Rocca & Sazima, 2008; Rocca & Sazima, 2010).

**Figure 1:**
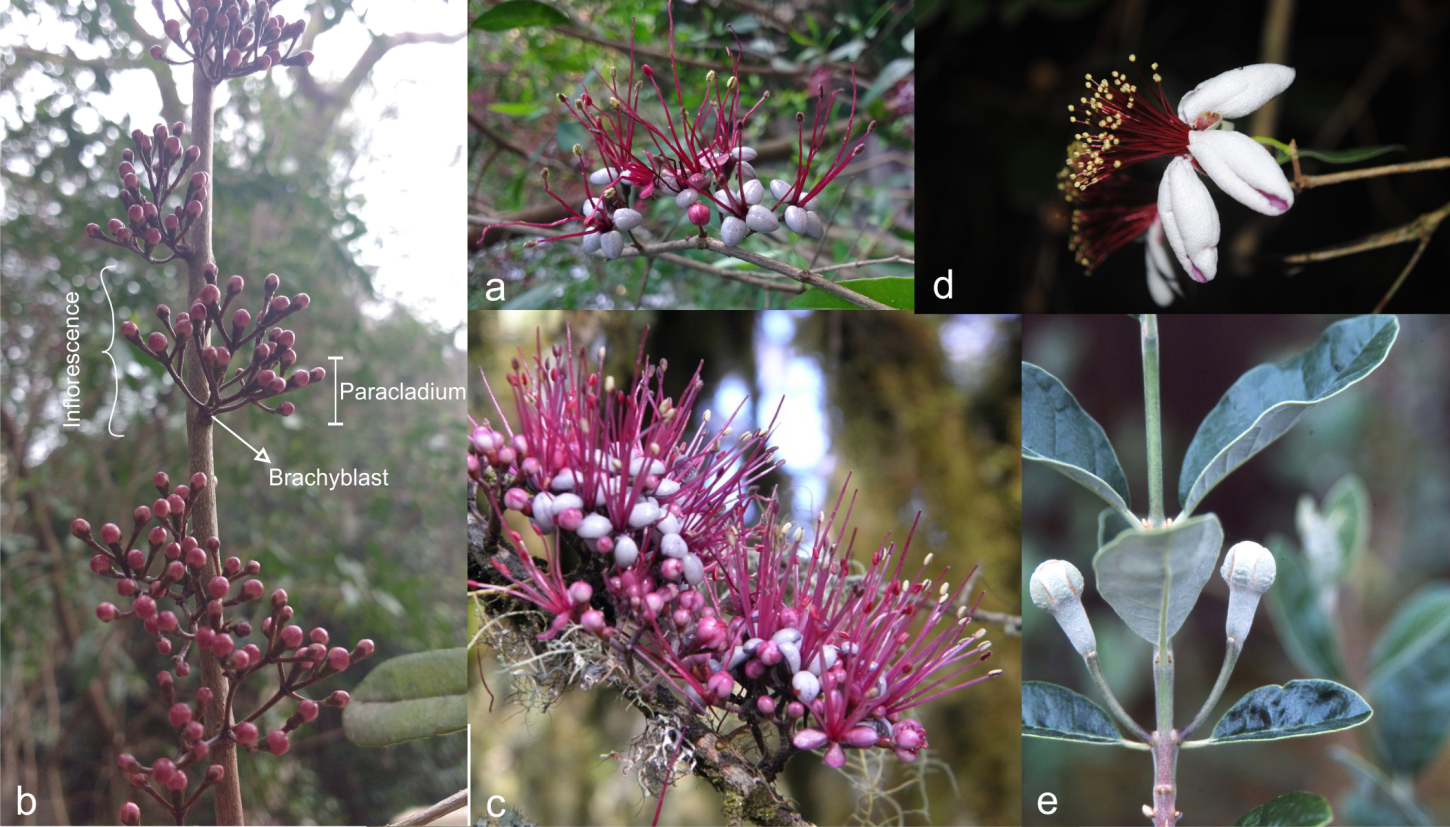
*Flowers and Inflorescences*. *Myrrhinium atrupurpureum* (A, B, C): (A) Flowers. (B) Cauliflorous inflorescences with closed flower buds; note the degree of branching of paracladia supported by each brachyblast. (C) Inflorescences with floral buds and open flowers; note the different degree of development and colour of petals and the typical appearance of brush-like inflorescence. *Acca sellowiana* (D, E): (D) Flowers. (E) Inflorescence with uniflorous paracladia in the axil of leaves. Photo credits: A and B, María Gabriela Nadra; C, Alfredo Grau, Universidad Nacional de Tucumán, Argentina; D, Andrés Gonzalez via http://floranativadeuruguay.blogspot.com.ar; E, Pat Breen, Oregon State University.

In the Myrtaceae family of the New World, the presence of nectar is uncommon and pollen is generally the main resource available to pollinators (Gressler, Pizo & Morellato, 2006). Myrtaceae comprises c. 5,500 currently recognized, extant species, classified in 140 genera distributed, with minor exceptions, in tropical and temperate regions of the southern-hemisphere continents. Fossils are also known from these continents and Antarctica (Poole, Hunt & Cantrill, 2001; Thornhill & Macphail, 2012; Vasconcelos et al., 2017). Bees are the most important group of pollinators of Myrtaceae (Lughadha & Proença, 1996). Bird pollination is often derived from bee pollination (and the reverse is less frequent; Thomson & Wilson, 2008); bird pollination is relatively common among Australian species of Myrtaceae, but it is only scarcely represented (<1% of species) among the New World members of the family (Roitman, Montaldo & Medan, 1997). The only cases of bird pollination reported among Myrtaceae native to the Americas are those of *Acca* O.Berg and *Myrrhinium* Schott, two small endemic South American genera with flowers bearing numerous red and robust stamens (Fig. 1), similar to those of many bird-pollinated Myrtaceae from Australasia (Lughadha & Proença, 1996). However, *Myrrhinium* and *Acca* are not pollinated by nectarivorous birds; both taxa, which produce neither nectar nor scent, offer fleshy petals as reward to be consumed by birds that otherwise eat fruits, not flower products; by these means, typically frugivorous birds act as effective pollinators in these plants (Roitman, Montaldo & Medan, 1997). Previous studies have reported that the flowers of *Acca sellowiana* (O.Berg) Burret are visited by fruit-eating birds, as well as by some insects (Popenoe, 1912; Mattos, 1986; Stewart, 1986; Stewart & Craig, 1989; Ducroquet & Hickel, 1997; Roitman, Montaldo & Medan, 1997; Hickel & Ducroquet, 2000; Degenhardt et al., 2001; Sazima & Sazima, 2007). Roitman, Montaldo & Medan (1997) studied in detail the pollination biology of *Myrrhinium atropurpureum* Schott, confirming that the main pollinators of this species are fruit and seed-eating birds. These are perching birds and consume the petals, thereby carrying pollen inadvertently on their heads and body to other flowers and trees.

Fleshy petals are among resources alternative to nectar and functional pollen reportedly offered by plants to pollinators, including tissues such as food scales, food bodies, pseudopollen, and other sweet tissues (Simpson & Neff, 1981). Floral or extrafloral food body rewards are functional in bird pollination, including the petals of *Myrrhinium* and *Acca* (Proctor, Yeo & Lack, 1996; Sersic & Cocucci, 1996; Dellinger et al., 2014). The petals in *Acca* and *Myrrhinium* differ in size, and the flowers differ greatly in their arrangement within inflorescences and other characters across species. *Acca* exhibits single large, multi-staminate flowers, each offering a copious pollen production, while the small flowers of *Myrrhinium* are clumped in dense cauliflorous inflorescences and show a marked reduction in the number of stamens, as compared to related genera (i.e., genera in the *Pimenta* group sensu Lucas et al., 2007). This reduction in per-flower pollen production, together with other features such as the compact arrangement of flowers, and changes in the coloration of the petals during anthesis, has been suggested to represent a greater tendency towards bird pollination in *Myrrhinium* as compared with *Acca* (Roitman, Montaldo & Medan, 1997).

Despite differences in degree of adaptation, whether this peculiar pollination interaction is unique, i.e., inherited from an ancestor common to *Acca* and *Myrrhinium*, or convergent in the two genera, remains unknown due to uncertainties in the systematics of Myrtae that we briefly revise here. The current classification of Myrtaceae proposed by Wilson et al. (2005) and Wilson (2011) recognizes two subfamilies and 17 tribes, including the tribe Myrteae to which both *Acca* and *Myrrhinium* belong (Australasian species pollinated by perching birds occur in other tribes, particularly Syzygieae, Eucalypteae, Melaleuceae, and Chamelaucieae). Myrteae DC. (sensu Wilson et al., 2005) is a pantropical group constituted by trees and shrubs (McVaugh, 1968; Lucas et al., 2007; Biffin et al., 2010; Vasconcelos et al., 2017). The tribe comprises 49 genera and 2,500 species and its diversity peaks in Central and South America (McVaugh, 1968; Govaerts et al., 2008). The tribe is distinguished from other closely related tribes mainly by the presence of fleshy fruits (Lucas et al., 2007). The phylogeny of Lucas et al. (2007), based on DNA sequence data, supported a division of the tribe into seven informal groups. One of them was the “*Pimenta* group” that contained *Acca* as well as five other genera (*Amomyrtus* (Burret) D. Legrand & Kausel, *Legrandia* Kausel*, Campomanesia* Ruiz & Pav., *Psidium* L.*,* and *Pimenta* Lindl.); *Myrrhinium* was not included in the analysis. Several authors have linked *Myrrhinium* to *Acca*, or to other genera in the *Pimenta* group (e.g., Kausel, 1956; Kausel, 1966; McVaugh, 1968). Landrum (1986) suggested that *Acca* and *Myrrhinium* are related, based on a shared set of floral features: tetramerous flowers, red, pink, or purple-coloured, exerted rigid stamens, and fleshy, sweet petals–all features very distinctive from what is commonly found in the *Pimenta* group. Also, Thornhill et al. (2012) observed that *Acca* and *Myrrhinium* share a pollen type slightly different from other Neotropical Myrteae. Vasconcelos et al. (2017) recently included *Myrrhinium* in a major phylogenetic analysis of Myrteae, but a level of uncertainty existed in regard to the position of this and other genera mainly due to the poor support of a *Pimenta* group that included *Acca* but not *Myrrhinium.* The later was recovered in a widely separated clade that placed *Myrrhinium* as sister to *Psidium*, in the Vasconcelos et al. (2017) *Psidium* group. Therefore, at present there are conflicting hypotheses of relationships among the genera relevant for the evolution of pollination by frugivorous birds in the tribe Myrteae.

Here we first generate a new, solid phylogenetic hypothesis that solves the position of the Neotropical ornithophilous genus within the Myrteae, building upon the efforts of Lucas et al. (2007), Biffin et al. (2010), and Vasconcelos et al. (2017), who successfully established the molecular basis for the classification of Myrteae. We revisit the systematic problem of the uncertain placement of Neotropical genera pollinated by frugivorous birds using more terminals in the groups of interest and contributing new sequences of *Myrrhinium* and other taxa. The newly generated sequences of *Myrrhinium* resolve the uncertainty around its phylogenetic position and allow for a strong test on the existence of a single evolutionary origin of this interaction in Myrteae. Second, we dissect the structure of flowers and inflorescences in *Acca*, *Myrrhinium*, and closely related genera in order to define a set of macroscopic characters relevant to assessing the fruit-bird ornitophily evolutionary problem. We mapped the characters onto our phylogeny and examine hypotheses of evolution of pollination by frugivorous birds in Myrteae.

## Materials & Methods

### Taxonomic sampling

We selected taxa following natural groupings established in Lucas et al. (2007), choosing a total of 86 terminals. We included 76 taxa considered to be part of the tribe Myrteae, 14 of which belong in the *Pimenta* group and may represent the closest relatives to *Acca* according to Lucas et al. (2007) and perhaps also *Myrrhinium*. Ten other taxa from different genera belonging to four other tribes of Myrtoideae (sensu Wilson et al., 2005) were also included. We followed Nixon & Carpenter (1993) in considering many outgroups as a general taxonomic sampling principle, and also because the positions of the *Pimenta* group members and other related taxa are not well resolved in previously published phylogenies. In particular, the position of *Acca sellowiana* is either unresolved or poorly supported. Our taxonomic sample thus represented 30 genera in Myrteae with four molecular markers (*mat*K, *psb*A-*trn*H, ITS/5.8S and ETS). Of these, 282 sequences were downloaded from GenBank (see Table S1 for accesion numbers) while 11 accesions were sequenced for the *mat*K, *psb*A-*trn*H, and ITS/5.8S markers for the present study (see Table S1 and below).

### DNA sequencing

Total genomic DNA was extracted from leaves of plants collected in the field and dried in silica gel using CTAB protocol (Doyle & Doyle, 1987). Three DNA regions were amplified: the plastid intergenic spacer *psb*A*-trn*H, the plastid *mat*K gene, and the nuclear ITS/5.8S region. These three regions were amplified by polymerase chain reaction (PCR).

The *mat*K gene was amplified using the primers matK 700F and matK 1710R of Gruenstaeudl et al. (2009) and Samuel et al. (2005) respectively. The plastid *psb*A*-trn*H spacer was amplified using the primers psbA and trnH of Hamilton (1999). The PCR for the *psb*A*-trn*H and *mat*K was carried out using the following parameters: one cycle of 94 °C for 5 min, 38 cycles of 94 °C for 30 s, 50 °C for 1 min, and 72 °C for 1 min, and a final extension cycle of 72 °C for 10 min. For the species that failed this protocol, variations in the annealing temperature (48–52 °C) were used. The ITS/5.8S region was amplified using the primers ITS4 and ITS5 of White et al. (1990) and the following PCR parameters: one cycle of 94 °C for 5 min, 38 cycles of 94 °C for 30 s, 58 °C for 1 min, and 72 °C for 1 min, and a final extension cycle of 72 °C for 10 min. For the species that failed this protocol, variations in the annealing temperature (56–58 °C) were used.

PCR reactions were performed in 25 µl final volumes with 50–100 ng of template DNA (concentration quantified with BioPhotometer©, Eppendorf, Hamburg, Germany), 0.2 µl of each primer, 25 µM dNTP, 5 mM MgCl2 1 × buffer and 0.3 units of Taq polymerase provided by Invitrogen Life Technologies. A negative control with no template was included for each series of amplifications to eliminate the possibility of contamination. PCR products were run out on a 1% TBE agarose gel stained with SYBR Safe DNA gel stain (Invitrogen) and visualized in a blue-light transilluminator. Automated sequencing was performed by Macrogen, Inc. The presence of a single peak corresponding to each nucleotide base was confirmed in all chromatograms; sequences with multiple peaks were discarded.

### Phylogenetic analyses

Sequences were aligned using the program Mafft 7 (Kantoh & Standley, 2013). The resulting alignment was checked and improved manually in BioEdit ver. 5.0.9 (Hall, 1999). The aligned matrix (Database S1) was submitted to TreeBASE (https://treebase.org/treebase-web/search/study/summary.html?id=22811). The *mat*K dataset included 73 sequences with an aligned length of 683 bp. The *psb*A*-trn*H dataset included 77 sequences and was 388 bp long when aligned. The ITS/5.8S alignment included 76 sequences of 688 bp. The ETS alignment included 67 sequences of 624 bp. Of these nucleotide positions, 11% (76 bp), 20% (76 bp), 27% (184 bp) and 33% (207 bp) were parsimony informative for the alignments of *mat*K, *psb*A-*trn*H, ITS/5.8S, and ETS, respectively. The datasets including the plastid and nuclear DNA sequences were analysed separately and in combination using Bayesian inference (BI) and maximum parsimony (MP).

The Akaike information criterion (AIC) implemented in jModeltest2 v2.1.6 (Darriba et al., 2012) selected the following models of nucleotide substitution per marker: TVM + G (*mat*K), TVM + I + G (*psb*A-*trn*H), TIM1 + G (ETS), TIM2 + I + G (ITS1), TIM3ef + I + G (5.8S) and TVM + I + G (ITS2). Bayesian inference analyses (BI) were conducted using MrBayes version 3.2.6 (Ronquist et al., 2012) through the CIPRES portal (Cyberinfrastructure for Phylogenetic Research) cluster at the San Diego Supercomputer Center (Miller, Pfeiffer & Schwartz, 2010). As applied to Bayesian analysis, we used the following models of nucleotide substitution: GTR + G for *mat*K and ETS, and GTR + I + G for *psb*A-*trn*H, ITS1, 5.8S and ITS2). The priors on state frequencies, rates and shape of the gamma distribution were estimated automatically from the data assuming no prior knowledge about their values (uniform Dirichlet prior). Four simultaneous analyses, starting from different random trees and with four Markov Monte Carlo chains were run for 15 million generations, sampling every 1,000 generations to ensure independence of the successive samples. The convergence and effective sample size were checked with the Average standard deviation of split frequencies (ASDSF) <0.01, the potential scale reduction Factor (PSRF) ∼1, and verifying with Tracer v. 1.6.0 (Rambaut et al., 2014) that effective sample size (ESS) for all parameters was over 300. The first 3,750 trees (25% of total trees) were discarded as burn-in; the remaining samples of each run were combined, and a Maximum Clade Credibility Tree (MCCT) was calculated using TreeAnnotator v1.8.3 (Drummond & Rambaut, 2007).

The maximum parsimony analyses were conducted using TNT ver. 1.1 (Goloboff, Farris & Nixon, 2008). All characters were equally weighted and treated as unordered. Gaps were scored as missing data. We used the option Driven Search which is especially indicated for large data matrices, set to find the minimum length 100 times with default settings for Sectorial Searches and Tree Fusing (Goloboff, 1999). All searches were done with random seed 0. The resulting trees were submitted to Ratchet (Nixon, 1999) and Tree Drifting (Goloboff, 1999), in both cases using default settings with 1,000 iterations. After the number of trees had stabilized, TNT was set to stop searching and the resulting trees were used to calculate a strict consensus tree. As a measure of clade stability, Jackknife (JK) values (see Farris et al., 1996) were calculated by means of 10,000 resampling iterations with a removal probability of 36%, using 10 replicates of Ratchet to find the minimum length once in each replication. Only JK values above 50% are reported. The Bremer support (BS) values (Bremer, 1994; Goloboff & Farris, 2001) were determined by sequencially search for trees 1–15 steps longer than optimal trees.

### Morphological analyses

Morphological studies were based on herbarium specimens deposited at Instituto de Botánica Darwinion, San Isidro, Buenos Aires, Argentina (SI; acronym follows Thiers, 2016) and specimens examined available from the authors. Five to ten herbarium sheets per species were analysed. We also examined fresh material of *Myrrhinium atropurpureum* var *octandrum* Benth. and *Acca sellowiana* cultivated at the garden of the Botanical Institute Darwinion and material collected in field in Tucumán Province, NW Argentina. Available literature on Myrtaceae inflorescences (Briggs & Johnson, 1979; Kawasaki, 1989; Landrum, 1986; Landrum, 1988a; Landrum, 1988b; Landrum & Donoso, 1990; Landrum & Kawasaki, 1997; Landrum & Salywon, 2004; Rotman, 1976a; Rotman, 1976b; Rotman, 1979; Rotman, 1986) was used when herbarium specimens were unavailable.

Thirty-two species belonging to the *Pimenta*, *Eugenia*, and *Myrteola* informal groups of Lucas et al. (2007) were included in the morphological study (see Table 1). The terminology used to describe the inflorescence’s architecture is based on Briggs & Johnson (1979), Weberling (1965), Weberling, Schwantes & Fleck (1981) and Rua (1999) unless otherwise stated.

**Table 1:** Morphological character matrix. Characters compiled for *Pimenta, Eugenia* and *Myrteola* groups (38 taxa) included in this study. Bibliographical references are indicated in the text.

**ESPECIES/CHARACTER**	**0**	**1**	**2**	**3**	**4**	**5**	**6**	**7**	**8**	**9**	**10**	**11**	**12**	**13**
*Acca_sellowiana*	0	1	–	–	[0 1]	[0 1]	1	0	15	1	2	13–24	1	1
*Amomyrtus_luma*	0	1	–	–	[0 1]	[0 1]	0	1	3	0	2	4–5	0	0
*Amomyrtus_meli*	[0 1]	1	0	–	[0 1]	[0 1]	0	1	3.5	0	2	5–7	0	0
*Campomanesia_guazumifolia*	0	1	–	–	0	0	0	1	15–20	0	4	5–15	0	0
*Eugenia_biflora*	0	1	–	–	[0 1]	[0 1]	0	0	–	0	2	3.5–5.5	0	0
*Eugenia_bimarginata*	0	1	–	–	1	1	0	–	–	0	–	–	0	0
*Eugenia_convexinervia*	0	1	–	–	0	0	0	–	–	0	2	–	0	0
*Eugenia_cuprea*	0	1	–	–	[0 1]	[0 1]	0	0	–	0	–	–	0	0
*Eugenia_dysenterica*	0	1	–	–	[0 1]	[0 1]	0	–	–	0	2	–	0	0
*Eugenia_sulcata*	0	1	–	–	[0 1]	[0 1]	0	0	7–9	0	2	–	0	0
*Eugenia_uniflora*	0	1	–	–	[0 1]	[0 1]	0	0	2.5–4	0	2	3–7	0	0
*Hexachlamys_edulis*	0	1	–	–	[0 1]	[0 1]	0	1	5–9	0	–	5-9	0	0
*Hexachlamys_hamiltonii*	0	1	–	–	[0 1]	[0 1]	0	–	–	0	–	–	0	0
*Hexachlamys_itatiaiensis*	0	1	–	–	[0 1]	[0 1]	0	–	–	0	–	–	0	0
*Legrandia_concinna*	0	1	–	–	0	0	0	0	7–10	0	4	7–12	0	0
*Lenwebbia_prominens*	0	1	–	–	0	0	0	0	2–6	0	[3 4]	3–7	0	0
*Lophomyrtus_bullata*	0	1	–	–	0	0	0	0	–	0	–	–	0	0
*Lophomyrtus_obcordata*	0	1	–	–	0	0	0	0	–	0	–	–	0	0
*Myrcianthes_cisplatensis*	[1 2]	1	0	–	0	0	0	1	–	0	2	3–5	0	0
*Myrcianthes_cisplatensis_11*	[1 2]	1	0	–	0	0	0	1	–	0	2	3–5	0	0
*Myrcianthes_fragrans*	[1 2]	1	0	–	0	0	0	0	–	0	2	–	0	0
*Myrcianthes_pseudomato*	[1 2]	1	0	–	0	0	0	0	5–7	0	2	6–10	0	0
*Myrcianthes_pseudomato_15*	[1 2]	1	0	–	0	0	0	0	5–7	0	2	6–10	0	0
*Myrcianthes_pungens*	0	1	–	–	0	0	0	0	7	0	3	9–11	0	0
*Myrcianthes_pungens_6*	0	1	–	–	0	0	0	0	7	0	3	9–11	0	0
*Myrrhinium_atropurpureum_1*	[3 4]	0	[0 1]	0	2	1	1	0	3.5–5	1	0	12–21	1	1
*Myrrhinium_atropurpureum_2*	[3 4]	0	[0 1]	0	2	1	1	0	3.5–5	1	0	12–21	1	1
*Myrteola_nummularia*	[0 1]	1	0	–	0	0	0	[0 1]	3.5	0	1	4–5	0	0
*Neomyrtus_pedunculata*	0	1	–	–	0	0	0	0	4–5	0	–	–	0	0
*Pimenta_dioica*	[3 4]	1	2	1	0	0	0	0	2	0	[2 3]	2–4	0	0
*Pimenta_pseudocaryophyllus*	3	1	2	1	0	0	0	0	3–5	0	[2 3]	4–6	0	0
*Pimenta_racemosa*	[3 4]	1	2	1	0	0	0	1	2	0	[2 3]	4–5	0	0
*Psidium_cattleianum*	0	1	–	–	0	0	0	[0 1]	5	0	–	5–7	0	0
*Psidium_friedrichsthalianum*	0	1	–	–	1	0	0	1	12	0	4	10–15	0	0
*Psidium_guajava*	0	1	–	–	[0 1]	[0 1]	0	[0 1]	13–22	0	4	7–15	0	0
*Psidium_guineense*	1	1	0	–	1	0	0	1	7–11	0	[3 4]	7–10	0	0
*Ugni_molinae*	[0 1]	1	0	–	0	0	0	[0 1]	5–8	1	2	2–4	0	0

We explored the morphology of flowers and inflorescences to identify characters that may compose the characters that are adaptive for fruit bird pollination. We developed 13 characters that could potentially contribute to identify changes for reconstructing the evolution of pollination by frugivorous birds in the group. We also coded the main pollination type, i.e., by bees versus by frugivorous bird as a fourteenth character. The 14 developed characters were the following: Branching degree of the paracladium (char.0), Development of apical meristem of paracladium (char. 1), Number of first order branching within the paracladium (char. 2), Complexity of the first order branches within the paracladium (char. 3), Elongation of internodes of the floriferous branches (char. 4), Type of foliage supporting the paracladia (char. 5), Fleshy petal presence (char. 6), Numbers of petals (char. 7), Length of petals (char. 8), Presence of pigments in petals (char. 9), Number of stamens (char. 10), Length of stamens (char. 11), Presence of purpureous pigments in filaments (char. 12) , Main pollination agent (char. 13).

The intraspecific variation observed was coded as double states for discrete characters. Multistate characters were considered additive. In addition, we coded two characters, length of petals (char. 8) and length of stamens (char. 11) as continuously varying characters scored as ranges. The character list and character definition is given in Suppl. File 2, and character codification in Table 1.

The characters were optimized onto the Bayesian consensus subtree containing the species of interest. We used parsimonious character reconstruction and continuous characters scored as ranges were optimized as such (for details on the optimization of continuous characters see Goloboff, Mattoni & Quinteros, 2006). All optimizations were done using the program TNT (Goloboff, Farris & Nixon, 2008). In addition, to provide statistics for character reconstruction we also performed the “Trace Character over Trees” routine implementing Parsimony in Mesquite v 3.50 (Maddison & Maddison, 2018). To account for topological uncertainty, the character state reconstruction was performed over a set of 2,000 trees resulting from the Bayesian analyses (last 500 trees per Markov chain). We did not reconstruct the optimization of the continuous characters with this method, as these are coded as ranges (not taken in Mesquite; to our knowledge, continuous characters coded as ranges can presently only be optimized in TNT).

## Results

### Phylogenetic analyses

Individual analyses of the four DNA datasets exhibited a poorly resolved tree topology (Fig. S1). The combined plastid and nuclear data matrix consisted of 86 terminals and 2,383 aligned characters with 23% of the nucleotide positions informative. Contrary to the individual marker analyses, the combined data matrix revealed considerable phylogenetic structure, described below.

**Figure 2:**
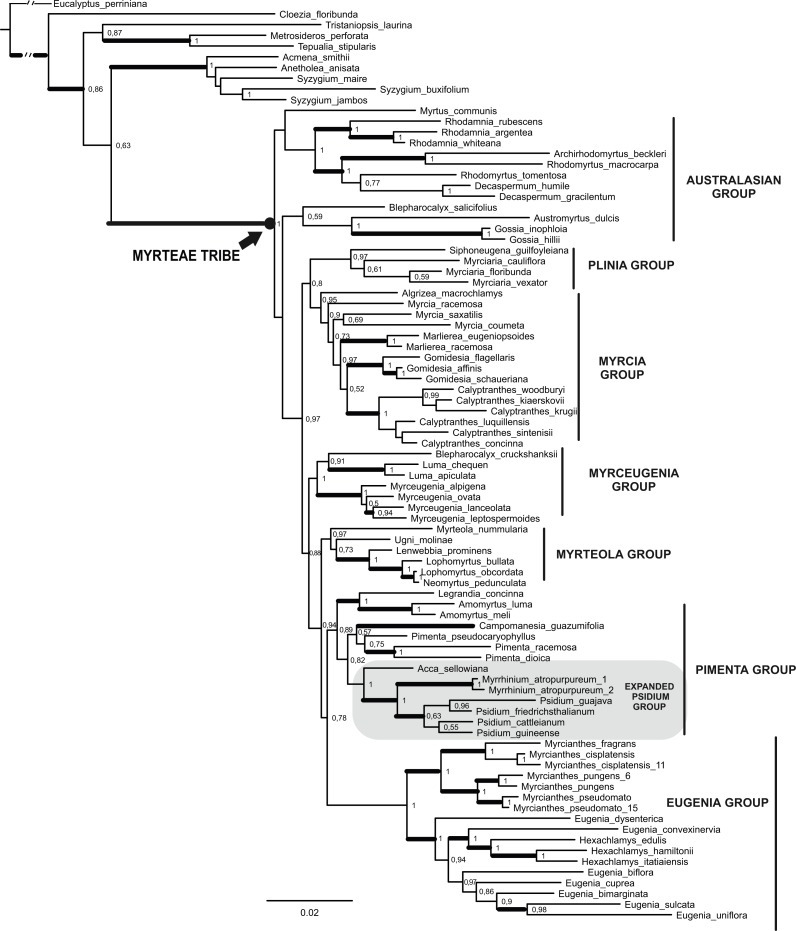
Maximum Clade Credibility Tree (from 45,000 trees) of the combined dataset (*mat*K, *psb*A*-trn*H, ITS/5.8 s and ETS) obtained by Bayesian Inference. Values on nodes correspond to Bayesian posterior probability (PP). Thick branches indicate >50 Jackknife support in parsimony analysis (see Fig. S2). Clade names sensu Lucas et al. (2007) except the expanded *Psidium* group (see text and Vasconcelos et al., 2017).

Parsimony analysis of the combined dataset resulted in 2,380 MP trees of 2,707 steps, with consistency index, CI = 0.483 and retention index, RI = 0.594 (as defined in Kluge, 1989; Kluge & Farris, 1969). The strict consensus tree is shown in Fig. S2. The BI analysis resulted in 45,000 trees summarized in the MCCT shown in Fig. 2. The MCCT of BI and the strict consensus parsimony tree were congruent.

In both BI and MP analyses, all species of Myrteae were grouped in a well-supported clade, with maximal (=1) posterior probabilities (PP), Bremer support (BS) = 12, and jackknife proportion JK = 100. Several distinct clades were identified within the tribe, and most informal groups suggested originally by Lucas et al. (2007) were also recovered in the present analyses. All groups were well supported, more than 0.89 PP, except the Australasian group that appeared polyphyletic. Our focal group *Pimenta* (sensu Lucas et al., 2007) was only recovered as monophyletic in the BI analysis. This *Pimenta* group of the BI analysis is composed of a clade joining *Legrandia* and *Amomyrtus* that is sister to the clades (*Pimenta* + *Campomanesia*) and (*Acca* + (*Psidium* + *Myrrhinium*)).

In all combined analyses both ornithophilous taxa*, Acca* and *Myrrhinium*, were found in the same clade, our expanded *Psidium* group (Fig. 2—cf. Vasconcelos et al., 2017), and phylogenetic relationships inside this group were compatible across the analyses presented here. In both analyses *Acca* was sister (PP = 1) to *Myrrhinium* + *Psidium* (PP = 1, JK = 94, BS = 3). *Psidium* was only monophyletic (but poorly supported) in the BI analysis while collapsed when using parsimony.

### Morphological character mapping

**Figure 3:**
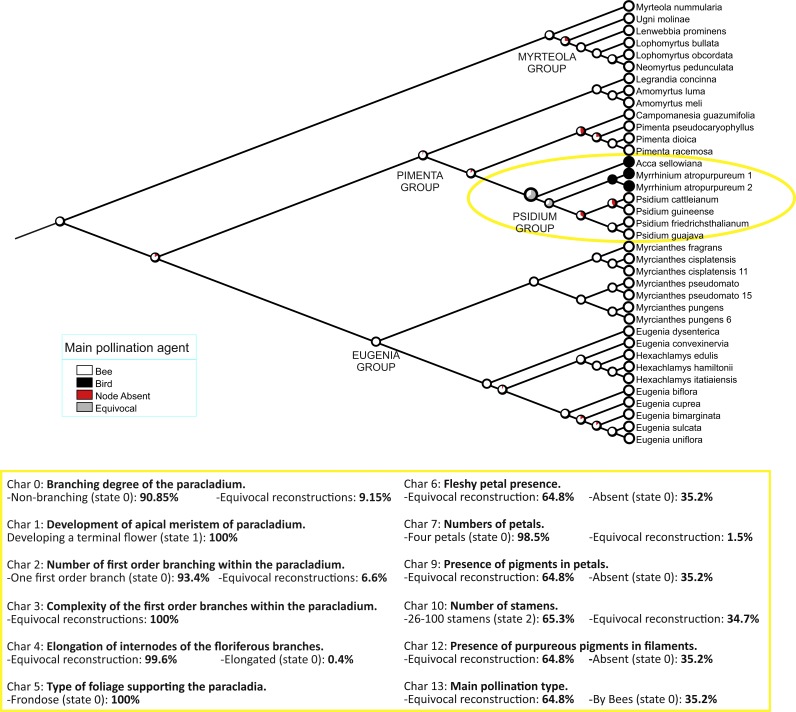
Character mapping. Mapping of main pollination type on 2,000 Bayesian subtrees (see ‘Methods’). Pie charts show the proportion of inferred states at each node. Below are the statistics of the remainder of characters for the ancestral node of the *Psidium* group (indicated in yellow on the tree).

**Figure 4:**
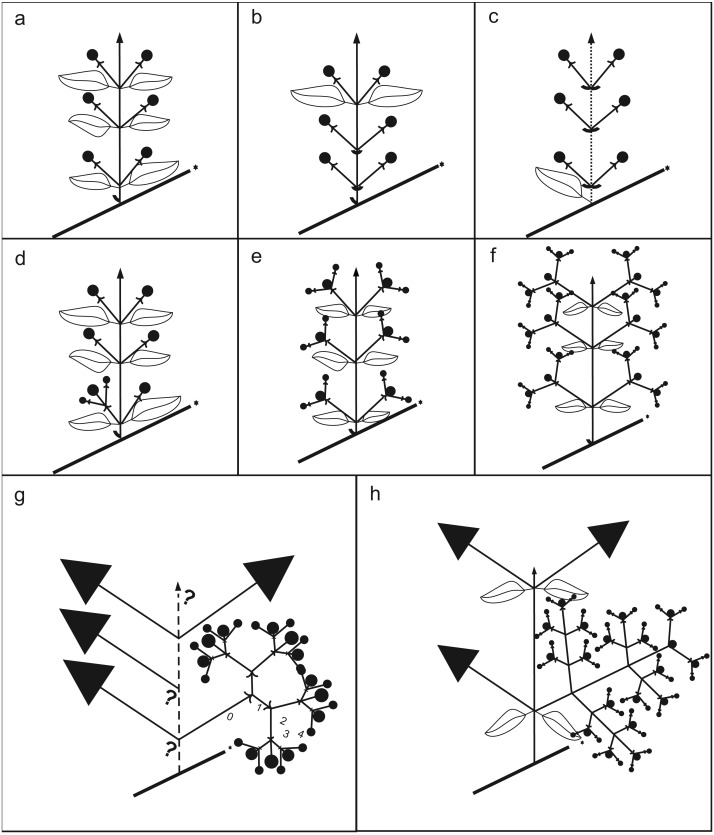
Schematic drawing of inflorescences. (A) Non-branching paracladium (branching degree of the paracladium 0; e.g., *Psidium guajava*). (B) Non-branching paracladium with foliose and bracteose leaves supporting the paracladia (e.g., *Acca*). (C) Non-branching paracladium, internode shortenings (dotted line; e.g., *Eugenia*, sometimes in *Acca*). (D) Branching degree of the paracladium 0 and first-order branching in the same plant (e.g., *Ugni, Myrteola*). (E) First-order branching (e.g., *Psidium guineense*). (F) Second order branching (e.g., *Myrcianthes*). (G) Third and fourth order branching, up to two homogeneous first order branches (e.g., *Myrrhinium*). (H) Third and fourth order branching, three or more first order branches with different degree of development, present only in *Pimenta*.

Optimization of our 14 characters, as codified in Table 1, is shown in Fig. 3 and Fig. S3. Changes specifically relevant to *Acca* and *Myrrhinium* are shown in Table 2 and inflorescences architecture types in Fig. 4. These analyses of inflorescence and floral architecture suggested that the ancestral inflorescence of the *Pimenta* group, and closely related genera, was a simple, unbranched inflorescence (char. 0, char. 2), with a single terminal flower (char. 1), supported by frondose leaves (char. 5) and elongate internodes (char. 4); see Fig. 4A. Mapping of inflorescence characters 0–5 indicated little or no change from ancestral states in *Acca* (Figs. 4A–4C and Appendix S1), but a greatly increased inflorescence complexity in *Myrrhinium*. The inflorescence in *Myrrhinium* (Fig. 4G and Appendix S1) evolved a larger number of paracladia, branched in complex ways (characters 0, 2, 3); the internodes were strongly reduced to form a brachyblast (character 4); and the foliose protection of each paracladium was reduced to bracts (character 5), which gave pollinators free access to the small fleshy petals of the individual flowers (Table 2).

**Table 2:** Character transformations in *Acca* and *Myrrhinium* as mapped in the Bayesian tree (Fig. 2). Character definition in the text. We report changes from ancestral states to states observed in each genus. For instance, the transformation 0–1 (arrow) represents the change from state 0 in the immediate ancestor to state 1 in the terminal, either *Acca* or *Myrrhinium*. No change is indicated with “=” (e.g., 0 = 0). Ambiguity is represented with “/” (e.g., 0/1 means state 0 or 1). Characters 8 (length of petals) and 11 (length of stamens) are continuous and states are expressed in mm.

Character	*Acca*	*Myrrhinium*	Interpretation of change
0	0 = 0	0 → 3/4	Increased degree of branching in *Myrrhinium*^a^
1	1 = 1	1 → 0	Truncated terminal of paracladia in *Myrrhinium*
2	NC	0 → 0/1	Increased number of first-order branching in *Myrrhinium*^a^
3	NC	0/1 → 0	Ambiguous in ancestor of *Myrrhinium*^b^
4	0/1 = 0/1	1 → 2	Internode shortening led to brachyblasts in *Myrrhinium*
5	0 → 0/1	0 → 1	Reduction of foliose protection of paracladia in *Myrrhinium*
6	01 → 1	01 → 1	Acquisition of fleshy petals in *Acca* and *Myrrhinium*
7	0 = 0	0 → 0	Ambiguous in ancestor of *Myrrhinium*
8	7 → 15	7 = 3.5–5	Lengthening (2X) of petals in *Acca*, no change (overlap with ancestor) in *Myrrhinium*
9	01 → 1	01 → 1	Pigmented (purpureous) petals^c^
10	2 → 2	2 → 0	Reduction of stamen number in *Myrrhinium*
11	10–12 → 13–24	10–12 → 12–21	Lengthening of stamens in *Acca* and *Myrrhinium*
12	01 → 1	01 → 1	Pigmented (purpureous) filaments in *Acca* and *Myrrhinium*

**Notes.**

NC state non-comparable in the terminal

a Convergent with *Pimenta*

b Differs from *Pimenta* (state 2)

c Convergent in *Ugni*

*Acca* and *Myrrhinium* primarily shared the presence of fleshy petals (char. 6). The mapping of this character onto our final Bayesian tree is ambiguous: either presence or absence of fleshy petals in the relevant ancestor of the *Psidium* group, inclusive of *Acca*, *Myrrhinium* and *Psidium* (see above). When this character is mapped independently onto the last 2,000 Bayesian trees (500 from each of four Markov chains), 1,296 reconstructions remain ambiguous and 704 favour petals that are not fleshy in the ancestor (see also Fig. 3). As expected, the characters with identical distribution (pigmentation of petals and stamens, and main pollination type) are also reconstructed in the same way.

Conservation or increase of flower display in *Acca* was reflected in the lengthening of petals (char. 8) and stamens (char. 11), and in the pigmentation of petals (char. 9) and staminal filaments (char. 12). The latter three character states are also present in *Myrrhinium*, but a reduction of individual-flower display in *Myrrhinium* was manifested in the shortening of petals (char. 8), and reduction of stamen number to just 4–8 (char. 10).

## Discussion

### Phylogenetic position of *Acca* and *Myrrhinium*

The MCCT tree and the strict consensus from the parsimony analysis were congruent, Myrteae was monophyletic and well-supported (1PP, 12 BS, 100 JK). Most informal groups suggested by Lucas et al. (2007) were also recovered here. Our focal group *Pimenta* (sensu Lucas et al., 2007) was recovered as monophyletic in the BI analysis, but not in the MP analysis, nor it was supported in the recent analysis of Vasconcelos et al. (2017).

Here, we recovered both ornithophilous taxa*, Acca* and *Myrrhinium*, in the same clade, our expanded *Psidium* group (Fig. 2—cf. Vasconcelos et al., 2017). *Acca* was sister to *Myrrhinium* + *Psidium*, with *Psidium* monophyletic in the BI analysis but collapsed when using parsimony. Thus, the ornithophilous taxa were recovered in the same group, as we expected, however, never as sister groups. The latter clade was a version of the *Psidium* group of Vasconcelos et al. (2017), here also including *Acca* in addition to *Psidium* and *Myrrhinium*.

This result differed notably from Vasconcelos et al. (2017), who found the former *Pimenta* group sensu Lucas et al. (2007) to be split into two distantly positioned groups, the *Pimenta* and *Psidium* groups, plus one isolated terminal, *Amomyrtus luma*. The *Pimenta* group included *Acca*, *Campomanesia*, *Legrandia*, *Pimenta*, and *Curitiba* in Vasconcelos et al. (2017), and their new *Psidium* group included *Myrrhynium*, *Psidium*, and *Mosiera* (the latter not included in our study). In the Maximum Likelihood and BI analyses from Vasconcelos et al. (2017), *Acca* was sister to *Campomanesia* in the *Pimenta* group but with low support, and *Psidium* was sister to *Myrrhinium* with high support; the latter grouping was also found in our results.

We argue that the different position of *Acca* in Vasconcelos et al. (2017) and in our study may reveal a nuclear vs. plastid conflict. This affects the perceived evolution of pollination by frugivorous birds because *Acca* and *Myrrhinium* are not closely related in Vasconcelos et al. (2017), but they belong in the same group here. The nuclear markers ITS/5.8S and ETS placed *Acca* in a group with *Psidium* and *Myrrhinium* in our study (Fig. S4), whereas *Acca* appeared distant from this group in our plastid phylogeny (using markers *mat*K and *psb*A-*trn*H; Fig. S5). *Acca* was in a group with *Psidium* in Biffin et al. (2010) using 18S–26S rDNA, ITS and 2 plastid markers and in Murillo, Stuessy & Ruiz (2013); Murillo, Stuessy & Ruiz (2016) using ITS/5.8S + ETS, but only in the ITS/5.8S tree of Vasconcelos et al. (2017); the latter authors used 8 plastid markers for their ITS/5.8S-plastid phylogeny in which the relationship of *Acca* and *Psidium* was lost, along with the link to *Myrrhinium*. Our hypothesis is that the nuclear evidence, here represented by ITS/5.8S and ETS, groups *Acca*, *Psidium*, and *Myrrhinium*, a grouping that is in conflict with at least part of the plastid evidence, that places *Acca* elsewhere. A nuclear-plastid conflict has been reported in the family Myrtaceae (Schuster et al., 2018) and in other large groups (e.g., in *Ficus*; Bruun-Lund et al., 2017). In our balance of evidence, we accept the result using our concatenated matrix and study the morphological characters on that topology (see below).

### Evolution of pollination by frugivorous birds in Myrteae

Our phylogenetic reconstruction of the tribe was essentially concatened with previous reports, except in details regarding the specific relationships of our focus taxa, *Acca* and *Myrrhinium*, which we place in the Myrteae phylogeny with an increased certainty. We found that the taxa do belong in the same group—our expanded *Psidium* group also inclusive of *Acca* (Fig. 2—cf. Vasconcelos et al., 2017). While more species of *Acca* and *Psidium* need to be included in future analyses, it is clear from the support values that we report, that the taxa pollinated by frugivorous birds are not sister to each other. Instead, *Acca* appeared as sister to *Myrrhinium* + *Psidium*, so either bird pollination originated at the basal node of our expanded *Psidium* group and was lost in *Psidium*, or it appeared in parallel in *Acca* and *Myrrhinium.*

Frugivorous birds visit *Acca* and *Myrrhinium* because they both offer fleshy sugary petals (Roitman, Montaldo & Medan, 1997). However, because the structural details of both flowers and inflorescences differ between the two genera we reconstructed the way the plant characters of this interaction evolved in each genus. This was done by mapping the 13 structural characters that we identified as relevant for understanding the evolution of the pollination by birds on the Bayesian subtree of Myrteae (see above). This subtree contained *Acca*, *Myrrhinium*, and related genera, the latter predominantly melitophilous (bee-pollinated) as in most Neotropical Myrtaceae (Lughadha & Proença, 1996); therefore, melittophily is the most likely ancestral pollination syndrome for the entire group (Lughadha & Proença, 1996). As expected, *Acca* and *Myrrhinium* acquired the same states in some characters along their evolutionary path to be pollinated by frugivorous birds. Surprisingly, they diverged greatly in other characters, suggesting that the interaction with fruit birds is modulated in ways idiosyncratic to each genus. The relatively little convergence with predominantly bee-pollinated myrtaceous genera (limited to particular character states also present in *Pimenta* and *Ugni*; see Table 2) strongly suggests that most character changes were directly related to the evolution of pollination by frugivorus birds, albeit the character states evolved quite differently in *Acca* and *Myrrhinium*. Specifically, both *Acca* and *Myrrhinium* converged in offering fleshy petals and attracting pollinators with a visual display of reddish or purpureous petals and stamens, the latter greatly elongated; however, the evolution of display differed between these taxa in important ways, as it appeared centred in enhancing traits of large individual flowers in *Acca*, and centred in the proliferation of small flowers in complex inflorescences in *Myrrhinium*.

Centring on flowers versus inflorescences for display may have consequences for the interaction with frugivorous birds, which often spend a very short time period during a particular visit to a plant (e.g., Blendinger et al., 2015). Because this interaction, from the plant perspective, consists of pollen transfer (export and reception), the display of one or few large flowers per inflorescence, each with many stamens, as in *Acca*, may facilitate massive pollen export. The many-flowered, brush-like, complex inflorescence in *Myrrhinium* may in turn facilitate successful pollen reception, as the visiting bird may contact many styles in one feeding bout. Interestingly, this does not necessarily compromise pollen export in this taxon, because stamens are few per flower but are available from the many flowers of the visited inflorescence. In addition, stamens in *Myrrhinium* are greatly elongated and much more exposed than in *Acca*. Whether these seemingly different strategies impact significantly in the pollination success of each species remains to be investigated. But previous authors, particularly Roitman, Montaldo & Medan (1997), have already pointed out differences between *Acca* and *Myrrhinium*, assigning a larger degree of adaptation to the latter. This suggestion is confirmed here in an explicit phylogenetic and evolutionary framework. These strategies rely upon the modular nature of flower parts and the inflorescence (see Niklas, 1994), which here seem to play a central role to enhance either or both adaptive functions of pollen export and import by means of repetition and subdivision of the variously nested modular components.

The presence of floral or extrafloral food bodies as reward for legitimate pollinators, whatever their nature, is extremely rare among flowering plants (Simpson & Neff, 1981). Dellinger et al. (2014) reported examples of food bodies offered by plants to birds; examples include the presence of edible bracts surrounding the flowers in Pandanaceae; glucose-rich corolla appendages in *Calceolaria* (Calceolariaceae); and the only known case of floral food-body reward associated with reproductive structures, the bulbous stamen appendages in *Axinaea* (Melastomataceae). The few examples compiled by Dellinger et al. (2014) also include the freshy petals found in *Acca* and *Myrrhinium*. Thus, the presence of edible petals as reward is extremely uncommon among angiosperms; in South America it has only been reported in the closely related myrtaceous genera *Myrrhinium* and *Acca* and in the unrelated *Calceolaria* (Roitman, Montaldo & Medan, 1997). This highlights the importance of this mutualism as a special case of evolution of plant-animal interactions.

## Conclusions

The South American myrtaceous genera *Acca* and *Myrrhinium* are known to share several characters (fleshy petals, reddish or purplish coloration and long stamens) that make them attractive as food bodies eaten by otherwise typical frugivorous birds. Our phylogenetic results indicate that *A. sellowiana* and *M. atropurpureum* are closely related and belong in the same group but they are not sister taxa but are successive sisters to a monophyletic *Psidium*—our expanded *Psidium* group. We cannot rule out a single evolutionary origin of some of the characters that appear ambiguous and may have contributed to the evolution of bird pollination in the common ancestor of our expanded *Psidium* group, which would be subsequently lost in species of the *Psidium* complex. This scenario may change with the inclusion of more related species in the phylogeny. *Acca* and *Myrrhinium* differ strikingly in other floral characters relevant for the interaction with fruit-eating birds and we suggest two strategies of flower exposure: large solitary flowers with numerous stamens, which maximize pollen export only (*Acca*), versus inflorescences with many small flowers, each with few, greatly exposed stamens, which simultaneously maximize both pollen reception and export functions (*Myrrhinium*). We thus confirm that *Myrrhinium*, as compared with *Acca*, evolved a greater degree of adaptation to be pollinated by frugivorous birds, and highlight the complexities that may be involved in the evolution of mutualistic plant-animal interactions.

##  Supplemental Information

10.7717/peerj.5426/supp-1 Table S1 GenBank accession numbers Accession numbers of 283 sequences downloaded from GenBank. Ten accessions sequenced for the *mat*K, *psb* A-*trn*H, and ITS/5.8S markers for the present study are in bold.

10.7717/peerj.5426/supp-2 Appendix S1 Morphological characters Definition of characters of the flower and inflorescence and character states examined in this study with explanatory comments. We optimize 13 morphological characters plus the character ”main pollination type”: 0–5 are characters of architecture of inflorescences and 6–12 characters of flowers. The numbers of each character and its states correspond to those presented in Table 1.

10.7717/peerj.5426/supp-3 Database S1 Aligned matrix *Mat*K, *psb*A-*trn*H, ITS/5.8S and ETS aligned matrix with new sequences generated in this work included.

10.7717/peerj.5426/supp-4 Figure S1 MCCT of four individual DNA datasets Individual analyses of the four DNA datasets (matK, *psb* A-*trn*H, ITS/5.8S and ETS).

10.7717/peerj.5426/supp-5 Figure S2 Strict consensus tree Strict consensus of 2,380 trees resulting from parsimony analysis (2,707 steps) of the combined dataset (*matK*, * psbA-trnH,* ITS/5.8S and ETS). Values on branches correspond to jackknife values above 50% (above the branch) and Bremer support values (below the branch).

10.7717/peerj.5426/supp-6 Figure S3 Mapping of morphological characters onto the Bayesian subtree Full definition of characters, and details of character states see in Appendix S1. Character 0: branching degree of the paracladium; character 1: development of apical meristem of paracladium; character 2: number of first order branching within the paracladium; character 3: complexity of the first order branches within the paracladium; character 4: elongation of internodes of the floriferous branches; character 5: type of foliage supporting the paracladio; character 6: fleshy petal presence; character 7: numbers of petals; character 8: length of petals; character 9: presence of pigments in petals; character 10: number of stamens; character 11: length of stamens ; character 12: presence of purpureous pigments in filaments; character 13: main pollination type.

10.7717/peerj.5426/supp-7 Figure S4 MCCT of the nuclear dataset Maximum Clade Credibility Tree (from 15,002 trees) of the nuclear dataset (ITS/5.8S and ETS) obtained by Bayesian Inference. Values on nodes correspond to Bayesian posterior probability (PP).

10.7717/peerj.5426/supp-8 Figure S5 MCCT of the plastid dataset Maximum Clade Credibility Tree (from 15,002 trees) of the plastid dataset (*mat*K and *psb*A-*tnr* H) obtained by Bayesian Inference. Values on nodes correspond to Bayesian posterior probability (PP).

## Additional Information and Declarations

### DNA Deposition

The following information was supplied regarding the deposition of DNA sequences: The DNA sequences for *Myrcianthes cisplatensis*, *Myrcianthes pseudomato*, *Myrcianthes pungens* and *Myrrhinium atropurpureum* var. *octandrum* are provided in the Supplemental Files.

### Data Availability

The following information was supplied regarding data availability: Accession numbers can be found in the Supplemental Information. TreeBASE: https://treebase.org/treebase-web/search/study/summary.html?id=22811.

## References

[ref-1] Armbruster WS, Patiny S (2011). Evolution and ecological implications of “specialized” pollinator rewards. Evolution of plant-pollinator relationships.

[ref-2] Biffin E, Lucas EJ, Craven LA, Da Costa IR, Harrington MG, Crisp MD (2010). Evolution of exceptional species richness among lineages of fleshy-fruited Myrtaceae. Annals of Botany.

[ref-3] Blendinger PG, Giannini NP, Zampini IC, Ordoñez R, Torres S, Sayago JE, Ruggera RA, Isla MI (2015). Nutrients in fruits as determinants of resource tracking by birds. Ibis.

[ref-4] Bremer K (1994). Branch support and tree stability. Cladistics.

[ref-5] Briggs BG, Johnson LAS (1979). Evolution in the Myrtaceae-evidence from inflorescence structure. Proceedings of the Linnean Society of New South Wales.

[ref-6] Bruun-Lund S, Clement WL, Kjellberg F, Rønsted N (2017). First plastid phylogenomic study reveals potential cyto-nuclear discordance in the evolutionary history of Ficus L.(Moraceae). Molecular Phylogenetics and Evolution.

[ref-7] Cronk Q, Ojeda I (2008). Bird-pollinated flowers in an evolutionary and molecular context. Journal of Experimental Botany.

[ref-8] Darriba D, Taboada GL, Doallo R, Posada D (2012). jModelTest 2: more models, new heuristics and parallel computing. Nature Methods.

[ref-9] Degenhardt J, Orth AI, Guerra MP, Ducroquet JP, Nodari RO (2001). Morfologia floral da goiabeira serrana (Feijoa sellowiana) e suas implicações na polinização. Revista Brasileira de Fruticultura.

[ref-10] Dellinger AS, Penneys DS, Staedler YM, Fragner L, Weckwerth W, Schönenberger J (2014). A specialized bird pollination system with a bellows mechanism for pollen transfer and staminal food body rewards. Current Biology.

[ref-11] Doyle JJ, Doyle JL (1987). A rapid procedure for DNA purification from small quantities of fresh leaf tissue. Phytochemical Bulletin.

[ref-12] Drummond AJ, Rambaut A (2007). BEAST: Bayesian evolutionary analysis by sampling trees. BMC Evolutionary Biology.

[ref-13] Ducroquet JPHJ, Hickel ER (1997). Birds as pollinators of Feijoa (Acca sellowiana Berg). Acta Horticulturae.

[ref-14] Farris JS, Albert VA, Källersjö M, Lipscomb D, Kluge AG (1996). Parsimony jackknifing outperforms neighbor-joining. Cladistics.

[ref-15] Faegri K, Van der Pijl L (1979). The principles of pollination ecology.

[ref-16] Fenster CB, Armbruster WS, Wilson P, Dudash MR, Thomson JD (2004). Pollination syndromes and floral specialization. Annual Review of Ecology, Evolution, and Systematics.

[ref-17] Goloboff PA (1999). Analyzing large data sets in reasonable times: solutions for composite optima. Cladistics.

[ref-18] Goloboff PA, Farris JS (2001). Methods for quick consensus estimation. Cladistics.

[ref-19] Goloboff PA, Farris JS, Nixon KC (2008). TNT, a free program for phylogenetic analysis. Cladistics.

[ref-20] Goloboff PA, Mattoni CI, Quinteros AS (2006). Continuous characters analyzed as such. Cladistics.

[ref-21] Govaerts R, Sobral M, Ashton P, Barrie F, Holst BK, Landrum LL, Matsumoto K, Mazine F, Lughadha N, Proenca C, Soares-Silva LH, Wilson PG, Lucas E (2008). World checklist of myrtaceae. The board of trustees of the Royal Botanic Gardens, Kew. published on the internet. http://apps.kew.org/wcsp/myrtaceae/.

[ref-22] Gressler E, Pizo MA, Morellato LPC (2006). Polinização e dispersão de sementes em Myrtaceae do Brasil. Brazilian Journal of Botany.

[ref-23] Gruenstaeudl M, Urtubey E, Jansen RK, Samuel R, Barfuss MH, Stuessy TF (2009). Phylogeny of Barnadesioideae (Asteraceae) inferred from DNA sequence data and morphology. Molecular Phylogenetics and Evolution.

[ref-24] Hall TA (1999). BioEdit: a user-friendly biological sequence alignment editor and analysis program for Windows 95/98/NT. Nucleic Acids Symposium Series.

[ref-25] Hamilton MB (1999). Four primer pairs for the amplification of chloroplast intergenic regions with intraspecific variation. Molecular Ecology.

[ref-26] Hickel ER, Ducroquet J (2000). Polinização entomófila da goiabeira serrana, Feijoa sellowiana (Berg). Revista Brasileira de Fruticultura.

[ref-27] Kantoh K, Standley DM (2013). MAFFT multiple sequence alignment software version 7: Improvements in perdormance and usability. Molecular Biology and Evolution.

[ref-28] Kausel E (1956). Beitrag zur Systematik der Myrtaceen. Almqvist & Wiksell.

[ref-29] Kausel E (1966). Lista de las Mirtáceas y Leptospermáceas argentinas. Lilloa.

[ref-30] Kawasaki ML (1989). Flora da Serra do Cipó, Minas Gerais: Myrtaceae. Boletim de Botânica Da Universidade de São Paulo.

[ref-31] Kluge AG (1989). A concern for evidence and a phylogenetic hypothesis of relationships among epicrates (Boidae, Serpentes). Systematic Biology.

[ref-32] Kluge AG, Farris JS (1969). Quantitative phyletics and the evolution of anurans. Systematic Biology.

[ref-33] Landrum LR (1986). Campomanesia, Pimenta, Blepharocalyx, Legrandia, Acca, Myrrhinium, and Luma (Myrtaceae). Flora Neotropica.

[ref-34] Landrum LR (1988a). Systematics of Myrteola (Myrtaceae). Systematic Botany.

[ref-35] Landrum LR (1988b). The myrtle family (Myrtaceae) in Chile. Proceedings of the California Academy of Sciences.

[ref-36] Landrum LR, Donoso C (1990). Ugni molinae (Myrtaceae), a potential fruit crop for regions of Mediterranean, maritime, and subtropical climates. Economic Botany.

[ref-37] Landrum LR, Kawasaki ML (1997). The genera of Myrtaceae in Brazil: an illustrated synoptic treatment and identification keys. Brittonia.

[ref-38] Landrum LR, Salywon A (2004). Systematics of Amomyrtus (Burret) D. Legrand & Kausel (Myrtaceae). Bonplandia.

[ref-39] Lucas EJ, Harris SA, Mazine FF, Belsham SR, Lughadha EN, Telford A, Chase MW (2007). Suprageneric phylogenetics of Myrteae, the generically richest tribe in Myrtaceae (Myrtales). Taxon.

[ref-40] Lughadha EN, Proença C (1996). A survey of the reproductive biology of the Myrtoideae (Myrtaceae). Annals of the Missouri Botanical Garden.

[ref-41] Maddison WP, Maddison DR (2018). http://www.mesquiteproject.org.

[ref-42] Mattos JR (1986). A goiabeira serrana. Publicação IPRNR 19.

[ref-43] McVaugh R (1968). The genera of American Myrtaceae: an interim report. Taxon.

[ref-44] Miller MA, Pfeiffer W, Schwartz T (2010). Creating the CIPRES Science Gateway for inference of large phylogenetic trees.

[ref-45] Murillo J, Stuessy TF, Ruiz E (2013). Phylogenetic relationships among Myrceugenia, Blepharocalyx, and Luma (Myrtaceae) based on paired-sites models and the secondary structures of ITS and ETS sequences. Plant Systamatics and Evololution.

[ref-46] Murillo J, Stuessy TF, Ruiz E (2016). Explaining disjunct distributions in the flora of southern South America: evolutionary history and biogeography of Myrceugenia (Myrtaceae). Journal of Biogeography.

[ref-47] Niklas KJ (1994). Plant allometry: the scaling of form and process.

[ref-48] Nixon KC (1999). The parsimony ratchet, a new method for rapid parsimony analysis. Cladistics.

[ref-49] Nixon KC, Carpenter JM (1993). On outgroups. Cladistics.

[ref-50] Norberg UM, Wainwright PC, Reilly SM (1994). Wing design, flight performance, and habitat use in bats. Ecological morphology: integrative organismal biology.

[ref-51] Poole I, Hunt RJ, Cantrill DJ (2001). A fossil wood flora from King George Island: ecological implications for an Antarctic Eocene vegetation. Annals of Botany.

[ref-52] Popenoe FW (1912). Feijoa sellowiana, its history, culture and varieties. Pomona College Journal of Economic Botany and Subtropical Horticulture.

[ref-53] Proctor MCF, Yeo P, Lack A (1996). The natural history of pollination.

[ref-54] Rambaut A, Suchard MA, Xie D, Drummond AJ (2014). http://beast.bio.ed.ac.uk/Tracer.

[ref-55] Rocca MA, Sazima M (2008). Ornithophilous canopy species in the Atlantic rain forest of southeastern Brazil. Journal of Field Ornithology.

[ref-56] Rocca MA, Sazima M (2010). Beyond hummingbird-flowers: the other side of ornithophily in the neotropics. Oecologia Australis.

[ref-57] Roitman GG, Montaldo NH, Medan D (1997). Pollination biology of Myrrhinium atropurpureum (Myrtaceae): sweet, fleshy petals attract frugivorous birds. Biotropica.

[ref-58] Ronquist F, Teslenko M, Van der Mark P, Ayres DL, Darling A, Höhna S, Larget B, Liu L, Suchard MA, Huelsenbeck JP (2012). MrBayes 3.2: efficient Bayesian phylogenetic inference and model choice across a large model space. Systematics Biology.

[ref-59] Rotman AD (1976a). Revisión del género Campomanesia en la Argentina (Myrtaceae). Darwiniana.

[ref-60] Rotman AD (1976b). Revisión del género Psidium en la Argentina (Myrtaceae). Darwiniana.

[ref-61] Rotman AD (1979). Las especies argentinas del género Myrcianthes (Myrtaceae). Darwiniana.

[ref-62] Rotman AD (1986). Las Myrtaceae del noroeste argentino. Darwiniana.

[ref-63] Rua GH (1999). Inflorescencias: bases teóricas para su análisis.

[ref-64] Samuel R, Kathriarachchi H, Hoffmann P, Barfuss MH, Wurdack KJ, Davis CC, Chase MW (2005). Molecular phylogenetics of Phyllanthaceae: evidence from plastid matK and nuclear PHYC sequences. American Journal of Botany.

[ref-65] Sazima I, Sazima M (2007). Petiscos florais: pétalas de Acca sellowiana (Myrtaceae) como fonte alimentar para aves em área urbana no Sul do Brasil. Biota Neotropica.

[ref-66] Schuster TM, Setaro SD, Tibbits JF, Batty EL, Fowler RM, McLay TGB, Wilcox S, Ades PK, Bayly MJ (2018). Chloroplast variation is incongruent with classification of the Australian bloodwood eucalypts (genus Corymbia, family Myrtaceae). PLOS ONE.

[ref-67] Sersic AN, Cocucci AA (1996). A remarkable case of ornithophily in Calceolaria: food bodies as rewards for a non-nectarivorous bird. Plant Biology.

[ref-68] Simpson BB, Neff JL (1981). Floral rewards: alternatives to pollen and nectar. Annals of the Missouri Botanical Garden.

[ref-69] Stewart P (1986). Birds, not bees for feijoas. Horticulture News.

[ref-70] Stewart AM, Craig JL (1989). Factors affecting pollinator effectiveness in Feijoa sellowiana. Journal of Crop and Horticultural Science.

[ref-71] Stiles FG (1981). Geographical aspects of bird-flower coevolution, with particular reference to Central America. Annals of the Missouri Botanical Garden.

[ref-72] Thiers B (2016). Index Herbariorum: a global directory of public herbaria and associated staff. New York Botanical Garden’s Virtual Herbarium. http://sweetgum.nybg.org/ih/.

[ref-73] Thomson JD, Wilson P (2008). Explaining evolutionary shifts between bee and hummingbird pollination: convergence, divergence, and directionality. International Journal of Plant Sciences.

[ref-74] Thornhill AH, Hope GS, Craven LA, Crisp MD (2012). Pollen morphology of the myrtaceae. part 4: tribes kanieae, myrteae and tristanieae. Australian Journal of Botany.

[ref-75] Thornhill AH, Macphail MK (2012). Fossil myrtaceous pollen as evidence for the evolutionary history of the Myrtaceae: a review of fossil Myrtaceidites species. Review of Palaeobotany and Palynology.

[ref-76] Vasconcelos TN, Proença CE, Ahmad B, Aguilar DS, Aguilar R, Amorim BS, Campbell K, Costa IR, De-Carvalho PS, Faria JEQ, Giaretta A, Kooij PW, Lima DF, Mazine FF, Peguero B, Prenner G, Santos MF, Soewarto J, Wingler A, Lucas E (2017). Myrteae phylogeny, calibration, biogeography and diversification patterns: increased understanding in the most species rich tribe of Myrtaceae. Molecular Phylogenetics and Evololution.

[ref-77] Weberling F (1965). Typology of inflorescences. Journal of the Linnean Society.

[ref-78] Weberling F, Schwantes HO, Fleck I (1981). Botánica sistemática: introducción a la botánica sistemática.

[ref-79] White TJ, Bruns T, Lee S, Taylor J, Innis MA, Gelfand DH, Shinsky JJ, White TJ (1990). Amplification and direct sequencing of fungal ribosomal RNA genes for phylogenetics. PCR protocols: a guide to methods and applications.

[ref-80] Wilson PG, Kubitzki K (2011). Myrtaceae. In ‘The families and genera of vascular plants. Vol. X Flowering plants Eudicots: Sapindales, Cucurbitales, Myrtaceae’.

[ref-81] Wilson PG, O’brien MM, Heslewood MM, Quinn CJ (2005). Relationships within Myrtaceae sensu lato based on a matK phylogeny. Plant Systematics and Evolution.

[ref-82] Zanata T, Dalsgaard B, Passos F, Cotton P, Ropper J, Maruyama PK, Fisher E, Schleuning M, Martín González AM, Vizentin-Bugoni J, Franklin D, Abrahamczyk S, Alarcon R, Araújo A, Araujo F, Azevedo-Júnior S, Baquero A, Böehning-Gaese K, Carstensen D, Chupil H, Coelho A, Faria R, Horak D, Ingcersen T, Janecek S, Kohler G, Las-Casas FM, Lopes A, Machado A, Machado CG, Machado IC, Maglianesi AM, Malucelli T, Mohd-Azlan A, Moura AC, Oliveira G, Oliveria PE, Ornelas JF, Riegert J, Rodrigues L, Lasprilla L, Rui AM, Sazima M, Schmid B, Sedlacek O, Timmermann A, Vollstädt M, Wang Z, Watts S, Rahbek C, Varassin IG (2017). Global patterns of interaction specialization in bird-flower networks. Journal of Biogeography.

