# Supplementary material for: Evolution of pollination by frugivorous birds in Neotropical Myrtaceae
Source: PeerJ. 2018 Aug 27;6:e5426. doi: 10.7717/peerj.5426 (PMC6118208; doi:10.7717/peerj.5426)
Supplement: Table S1 — Accession numbers of 283 sequences downloaded from GenBank. Ten accessions sequenced for the matK, psb A-trnH, and ITS/5.8S markers for the present study are in bold. [file peerj-06-5426-s001.docx]

*Acca sellowiana (0. Berg) Burret* ( AM489973.1/AM489807.1/AM234067.1/AM489888.1 )

*Algrizea macrochlamys (DC.) Proença & NicLugh.* ( AM489975.1/AM489809.1/AM234126.1/AM489890.1 )

*Amomyrtus luma (Molina) D.Legrand & Kausel* ( KM065323.1/AM489811.1/AM234073.1/AM489892.1 )

*Amomyrtus meli (Phil.) D.Legrand & Kausel* ( AM489976.1/AM489810.1/AM234069.1/AM489891.1 )

*Archirhodomyrtus beckleri (F.Muell.) A.J.Scott* ( AF368197.1/KM895175.1/HQ225435.1/)

*Austromyrtus dulcis (C.T.White) L.S.Sm.* ( AM489977.1/AM489813.1/AM234133.1/AM489894.1 )

*Blepharocalyx cruckshanksii (Hook. & Arn.) Nied.* ( AM489978.1/AM489814.1/AM234070.1/AM489895.1 )

*Blepharocalyx salicifolius (Kunth) O.Berg* ( AM489979.1/AM489815.1/AM234084.1/AM489896.1 )

*Calyptranthes concinna DC.* ( AM489980.1/AM489817.1/AM234103.1/AM489898.1 )

*Calyptranthes kiaerskovii Krug & Urb.* ( AM489981.1/AM489819.1/AM234105.1/AM489900.1 )

*Calyptranthes krugii Kiaersk.* ( KJ012496.1/KJ426640.1/-/- )

*Calyptranthes luquillensis Alain* ( KJ012497.1/-/-/- )

*Calyptranthes sintenisii Kiaersk.* ( KJ012498.1/KJ426643.1/-/- )

*Calyptranthes woodburyi Alain,* ( KJ012499.1/KJ426644.1/-/- )

*Campomanesia guazumifolia (Cambess.) O.Berg* ( AY521532.1/AM489821.1/AM234076.1/AM489902.1 )

*Cloezia floribunda Brongn. & Gris* ( AY521533.1/-/AF172767.1/AY606255.1 )

*Decaspermum gracilentum (Hance) Merr. & L.M.Perry* ( KJ510961.1/-/-/- )

*Decaspermum humile (Sweet ex G.Don) A.J.Scott* ( AY521534.1/AM489824.1/AM234128.1/AM489905.1 )

*Eucalyptus perriniana F.Muell. ex Rodway* ( AM489985.1/AM489825.1/AM234139.1/AM489907.1 )

*Eugenia biflora (L.) DC.* ( KJ012585.1/KJ469659.1/KJ187610.1/KJ187663.1 )

*Eugenia bimarginata DC.* ( -/KJ469660.1/KJ187611.1/KJ187664.1 )

Eugenia convexinervia D.Legrand ( -/KJ469667.1/KJ187618.1/KJ187670.1 )

*Eugenia cuprea (O.Berg) Nied. in H.G.A.Engler & K.A.E.Prantl* ( -/KJ469668.1/KJ187619.1/KJ187671.1 )

*Eugenia dysenterica DC*. ( J850043.1/KJ469669.1/KJ187620.1/KJ187672.1 )

*Eugenia sulcata Spring ex Mart.* ( AM489987.1/AM489829.1/KJ187647.1/KJ187701.1 )

*Eugenia uniflora L*. ( AM489986.1,/AM489828.1/AM234088.1/JF682795.1 )

*Gomidesia flagellaris D.Legrand* ( AM489989.1/AM489836.1/AM234113.1/AM489918.1 )

*Gomidesia affinis (Cambess.) D.Legrand* ( KF981337.1/AM489834.1/AM234111.1/AM489916.1 )

*Gomidesia schaueriana O.Berg in C.F.P.von Martius & auct. suc. (eds.)* (AM489988.1/AM489835.1/AM234112.1/AM489917.1 )

*Gossia hillii (Benth.) N.Snow & Guymer* ( -/AM489838.1/AM234132.1/AM489920.1 )

*Gossia inophloia (J.F.Bailey & C.T.White) N.Snow & Guymer* ( -/AM489837.1/AM234131.1/AM489919.1 )

*Hexachlamys edulis (O.Berg) Kausel & D.Legrand* ( AY525131.1/KJ469702.1/KJ187652.1/KU945982.1 )

*Hexachlamys hamiltonii Mattos* ( -/KJ469703.1/KJ187653.1/KJ187706.1 )

*Hexachlamys itatiaiensis var. kleinii D.Legrand ex Mattos* ( -/KJ469704.1/KJ187654.1/KJ187707.1 )

*Legrandia concinna (Phil.) Kausel* ( AM489990.1/AM489839.1/AM234072.1/AM489921.1 )

*Lenwebbia prominens N.Snow & Guymer* ( AY521538.1/KM895153.1/-/- )

*Lophomyrtus bullata Burret, Notizbl.* ( AM489992.1/AM489841.1/AM234145.1/AM489923.1 )

*Lophomyrtus obcordata (Raoul) Burret* ( AM489993.1/AM489842.1/AM234146.1/AM489924.1 )

*Luma apiculata (DC.) Burret* ( AM489995.1/AM489843.1/AM234101.1/AM489926.1 )

*Luma chequen (Molina) A.Gray* ( JN661010.1/AM489844.1/AM234102.1/AM489927.1 )

*Marlierea eugeniopsoides (D.Legrand & Kausel) D.Legrand* (AM489996.1/AM489845.1/AM234107.1/AM489928.1 )

*Marlierea racemosa (Vell.) Kiaersk.* ( JN091303.1/JN091398.1/JN091207.1/JN091259.1 )

*Metrosideros perforata (J.R.Forst. & G.Forst.) Druce*( AM489998.1/AM489848.1/AM234141.1/AM489931.1 )

*Myrceugenia alpigena (DC.) Landrum* ( JN660991.1/AM489854.1/AM234098.1/AM489937.1 )

*Myrceugenia lanceolata* (Juss. ex J.St.-Hil.) Kausel ( JN661007.1/AM489849.1/AM234074.1/AM489932.1 )

*Myrceugenia leptospermoides* (DC.) Kausel ( AM489999.1/AM489850.1/AM234075.1/AM489933.1 )

*Myrceugenia ovata (Hook. & Arn.) O.Berg* ( JN661021.1/AM489852.1/AM234096.1/AM489935.1 )

*Myrcia coumete (Aubl.) DC.* ( GQ248163.1/GQ248348.1/AM234123.1/AM489947.1 )

*Myrcia racemosa (O.Berg) Kiaersk.* ( AM490005.1/AM489861.1/KP722380.1/AM489944.1 )

*Myrcia saxatilis (Amshoff) McVaugh* ( AM490004.1/AM489860.1/AM234119.1/AM489943.1 )

*Myrcianthes cisplatensis* (**MH713605**/**MH713604**/**MH716438**/- )

*Myrcianthes pseudomato* (**MH713606**/-/**MH716439**/- )

*Myrcianthes pungens* ( -/-/**MH716440**/- )

*Myrcianthes cisplatensis (Cambess.) O.Berg* ( -/JQ033349.1/JN660914.1/JN660864.1 )

*Myrcianthes fragrans (Sw.) McVaugh* ( KJ772955.1/KJ469705.1/KJ187655.1/KJ187708.1 )

*Myrcianthes pseudomato (D.Legrand) McVaugh* ( -/AM489868.1/AM234100.1/AM489951.1 )

*Myrcianthes pungens (O.Berg) D.Legrand* ( -/AM489867.1/KJ187656.1/KJ187709.1 )

*Myrciaria cauliflora (Mart.) O.Berg* ( AM490007.1/AM489869.1/AM234093.1/AM489952.1 )

*Myrciaria floribunda (H.West ex Willd.) O.Berg* ( -/KJ426839.1/AM234094.1/AM489953.1 )

*Myrciaria vexator McVaugh* ( AY521544.1/-/-/- )

*Myrrhinium atropurpureum var. octandrum Benth.*1 ( -/**MH713602**/**MH716441**/- )

*Myrrhinium atropurpureum var. octandrum Benth.* 2 (**MH713607**/**MH713603**/**MH716442**/- )

*Myrteola nummularia (Lam.) O.Berg* ( AM490008.1/AM489871.1/AM234068.1/AM489954.1 )

*Myrtus communis L*. ( AM490009.1/AM489872.1/AM234149.1/AM489955.1 )

*Neomyrtus pedunculata* (Hook.f.) Allan ( AM490010.1/AM490637.1/AM234144.1/AM489956.1 )

*Pimenta dioica (L.) Merr.* ( AM490011.1/AM489874.1/AM234081.1/AM489958.1 )

*Pimenta pseudocaryophyllus (Gomes) Landrum* ( AM490013.1/AM489876.1/AM234083.1 /AM489960.1 )

*Pimenta racemosa (Mill.) J.W.Moore* ( AM490012.1/AM489875.1/AM234082.1/AM489959.1 )

*Psidium cattleyanum Afzel. ex Sabine* ( AM490014.1/AM489878.1/AM234080.1/AM489962.1 )

*Psidium friedrichsthalianum (O.Berg) Nied* ( GQ982073.1/GQ982335.1/-/- )

*Psidium guajava L*. ( JQ024987.1/GQ434986.1/AY864898.1/AY454126.1 )

*Psidium guineense Sw.* ( JQ588513.1 /HG963888.1/-/- )

*Rhodamnia argentea Benth.* ( AF368217.1/AM489880.1/AM234129.1/AY454149.1 )

*Rhodamnia rubescens (Benth.) Miq.* ( AM490015.1/AM489879.1/AM234127.1/AM489963.1 )

*Rhodamnia whiteana Guymer & Jessup* ( JN564155.1/KM895192.1/-/- )

*Rhodomyrtus macrocarpa Benth.* ( AY525137.1/-/HQ225456.1/- )

*Rhodomyrtus tomentosa (Aiton) Hassk*. ( HQ415361.1/HQ415540.1/AF105085.1/- )

*Siphoneugena guilfoyleiana Proença* ( AM490016.1/AM490638.1/AM234085.1/AM489966.1 )

*Syzygium anisatum (Vickery) Craven & Biffin (* AF368195.2/AM489812.1/AM234138.1/AM489893.1 )

*Syzygium buxifolium Hook. & Arn*. ( HQ427387.1/HQ427087.1/EF026624.1/- )

*Syzygium jambos (L.) Alston* ( DQ088583.1/AM489882.1/EF026629.1/AM489967.1 )

*Syzygium maire (A.Cunn.) Sykes & Garn.-*Jones ( DQ088589.1/AM489883.1/EF026632.1/AM489968.1 )

*Syzygium smithii (Poir.) Nied* ( DQ088545.1/AM489808.1/AM234137.1/AM489889.1 )

*Tepualia stipularis (Hook. & Arn.) Griseb.* ( AF368222.2/AM489884.1/AM234071.1/AM489969.1 )

*Tristaniopsis laurina (Sm.) Peter G.Wilson & J.T.Waterh.* ( AF184710.2// KM064872.1/KU945985.1 )

*Ugni molinae Turcz.* ( AM490018.1//AM489885.1//AM234143.1/AM489970.1 )
