# Supplementary material for: Evolution of pollination by frugivorous birds in Neotropical Myrtaceae
Source: PeerJ. 2018 Aug 27;6:e5426. doi: 10.7717/peerj.5426 (PMC6118208; doi:10.7717/peerj.5426)
Supplement: Appendix S1 — Definition of characters of the flower and inflorescence and character states examined in this study with explanatory comments. We optimize 13 morphological characters plus the character ”main pollination type”: 0–5 are characters of architecture of inflorescences and 6–12 characters of flowers. The numbers of each character and its states correspond to those presented in Table 1. [file peerj-06-5426-s002.docx]

**Morphological characters**

Definition of characters of the flower and inflorescence and character states examined in this study with explanatory comments. We optimize 13 morphological characters plus the character "main pollination type": 0 to 5 are characters of architecture of inflorescences and 6 to 12 characters of flowers. The numbers of each character and its states correspond to those presented in Table 1.

**Character 0: Branching degree of the paracladium.** State 0: non-branching, paracladium with a single terminal flower; state 1: first-order branching; state 2: second order branching; state 3: third order branching; state 4: fourth order branching.

Most of the studied taxa presented inflorescences with non-branching paracladia (state 0) or with a first order branching only (state 1). Among these taxa are *Acca* (Fig. 4b), *Psidium (*Fig. 4 a, d, e) and *Eugenia* (Fig.4 a, c). First and second order branching paracladia were observed in *Myrcianthes (*Fig. 4 e,f). The most complex inflorescences were found in *Myrrhinium* (Fig. 4 g) and *Pimenta* species (Fig. 4 h), which exhibited paracladia branched 3 to 4 times.

**Character 1: Development of apical meristem of paracladium.** State 0: development truncated; state 1: apical meristem of paracladium developing a terminal flower.

All the taxa examined had paracladia whose main axis developed into a terminal flower. The only exception was *Myrrhinium*, in which the axes of the paracladia develop a terminal flower only exceptionally and are usually truncated Fig. 4 g.

**Character 2: Number of first order branching within the paracladium.** State 0: one first order branch; state 1: two first order branches; state 2: three or more first order branches.

All taxa with branched paracladia (i.e., non-uniflorous) had a single first-order branch, with the exception of *Myrrhinium* (state 0 and 1) and *Pimenta* (state 2) that in most cases had three or more branches of first order within the paracladia (Fig. 4 h). Non-comparable in uniflorous terminals (e.g., *Eugenia*).

**Character 3: Complexity of the first order branches within the paracladium.** State 0: Homogeneous branching pattern of first order branch; state 1: different degree of branching of first order branches.

This character separates *Pimenta* from the remaining genera. *Pimenta* has several successive first-order branches along the main axis of the paracladium. These first-order branches have a higher degree of branching in the proximal branches while the degree of branching is reduced in the more distal braches, producing a panicle (Fig. 4 h). *Myrrhinium* may have two first order branches, but with homogeneous branching pattern.

**Character 4: Elongation of internodes of the floriferous branches.** State 0: elongated; state 1: condensed; state 2: synflorescence forming a brachyblast.

In *Myrrhinium* the synflorescence forms a condensed brachyblast in which the internodes of the floriferous branch are extremely reduced (Fig. 4 g). These brachyblast support several paracladia. Similar structures were found in no other taxa, although some species presented internode shortenings (state 1) in some regions (e.g., *Eugenia,* Fig. 4 d). *Acca* occasionally presents an accumulation of paracladia caused by a reduction in length of internodes at the extremities of old branches (state 1).

**Character 5: Type of foliage supporting the paracladia.** State 0: frondose; state 1: bracteose.

Nearly all taxa present state 0, specifically a leaf supporting the paracladia. *Myrrhinium* is the only exception (state 1), with bracts always supporting the paracladia in the brachyblast (Fig. 4 g). In some species, both character states appear on the same plant, e.g., *Acca* in which the leaves of inflorescence can be frondose, bracteosa (see Fig. 4 c) or of intermediate aspect.

**Character 6: Fleshy petal presence.** State 0: absent; state 1: present.

This character is only present in *Acca* and *Myrrhinium* and represents the nutritional basis of fruit-bird ornithophily (Fig. 1 a, c, d).

**Character 7: Numbers of petals.** State 0: four petals; state 1: five petals.

Some species have 4 or 5 petals, even in the same plant, while most have a fixed number of petals. In both *Myrrhinium* and *Acca* the flowers bear 4 petals (Fig. 1a, d).

**Character 8: Length of petals.** Coded as continuously varying character, measured in mm.

*Acca*, *Psidium* and *Campomanesia* have large petals. The remaining species tend to have smaller petals, and among these, *Myrrhinium* has the smallest petals (≤5 mm).

**Character 9: Presence of pigments in petals.** State 0: absent (white); state 1: present. All taxa exhibit white petals, except *Acca*, *Myrrhinium*, and *Ugni*. In *Acca*, the abaxial surface is white while the adaxial surface is pink or reddish (Fig. 1 d). In contrast, *Myrrhinium* possess the adaxial surface pearly white and the abaxial surface is the one reddish or purplish (Fig. 1 a).

**Character 10: Number of stamens.** State 0: up to 9 stamens; state 1: 10-25 stamens; state 2: 26-100 stamens; state 3: 101-200 stamens; state 4: >200 stamens.

Some taxa have numerous stamens (states 3 or 4), such as *Pimenta*, *Psidium*, *Legrandia*, and *Campomanesia*. However, most taxa present an intermediate number of stamens (e.g., up to 100), among which is *Acca* (Fig. 1 d). *Myrteola* presented up to 25 stamens, while *Myrrhinium* (Fig. 1 a) differ from all other genera by having just 4 to 8 stamens (state 0).

**Character 11: Length of stamens.** Coded as continuously varying character, measured in mm.

*Acca* and *Myrrhinium* stand out from the rest of the taxa by having very long stamens (up to 24 mm). Some species of *Psidium*, *Pimenta*, and *Campomanesia* present stamens up to 15 mm long, while the remaining genera have shorter stamens (<10 mm).

**Character 12: Presence of purpureous pigments in filaments.** State 0: absent; state 1: present. Red or purplish filaments are only present in *Acca* and *Myrrhinium* (Fig. 1 a, c, d).

**Character 13: Main pollination type.** State 0: by bees; state 1: by birds.
