# Supplementary figures and images for: Evolution of pollination by frugivorous birds in Neotropical Myrtaceae
Source: PeerJ. 2018 Aug 27;6:e5426. doi: 10.7717/peerj.5426 (PMC6118208; doi:10.7717/peerj.5426)

matK

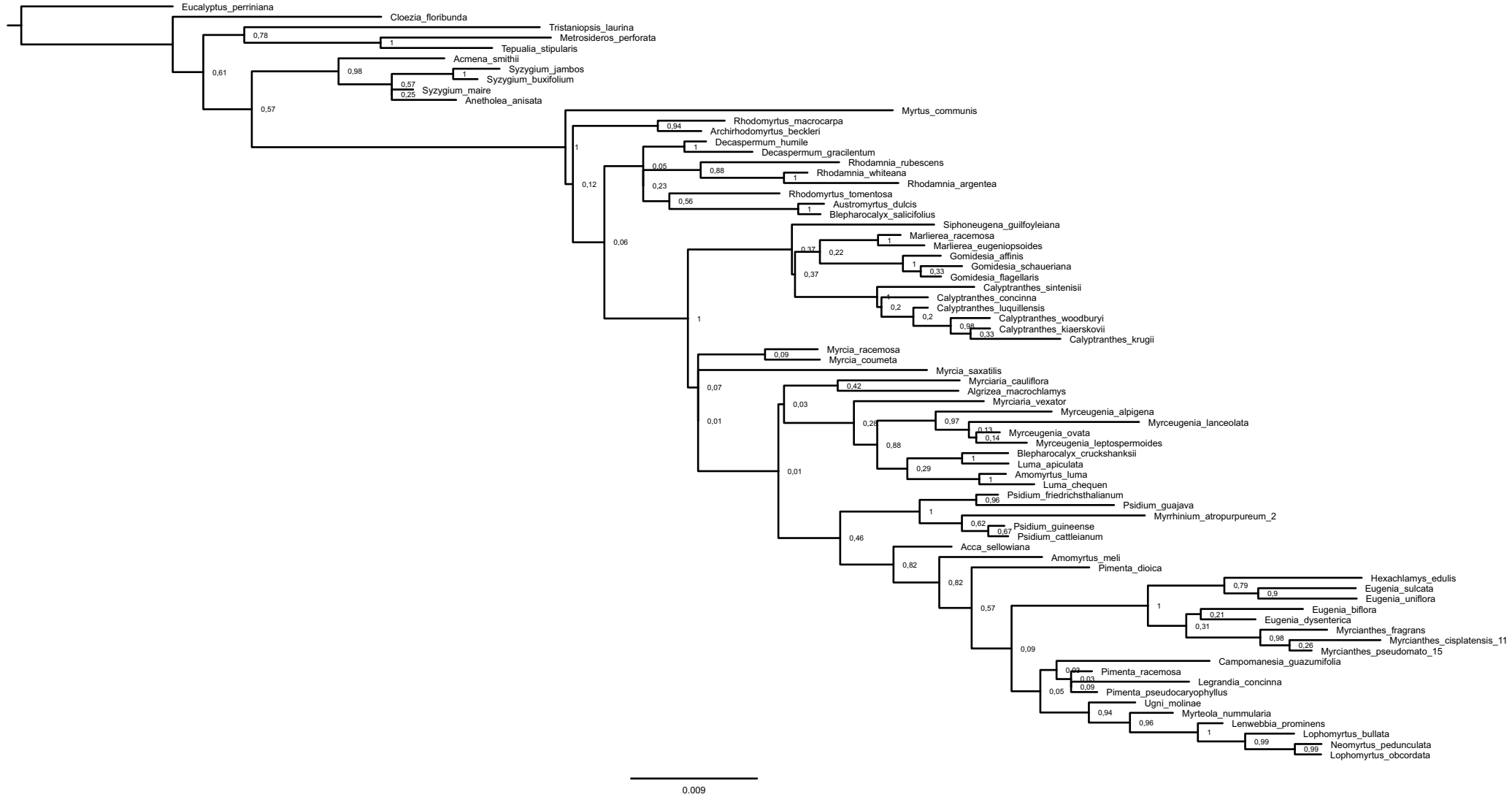

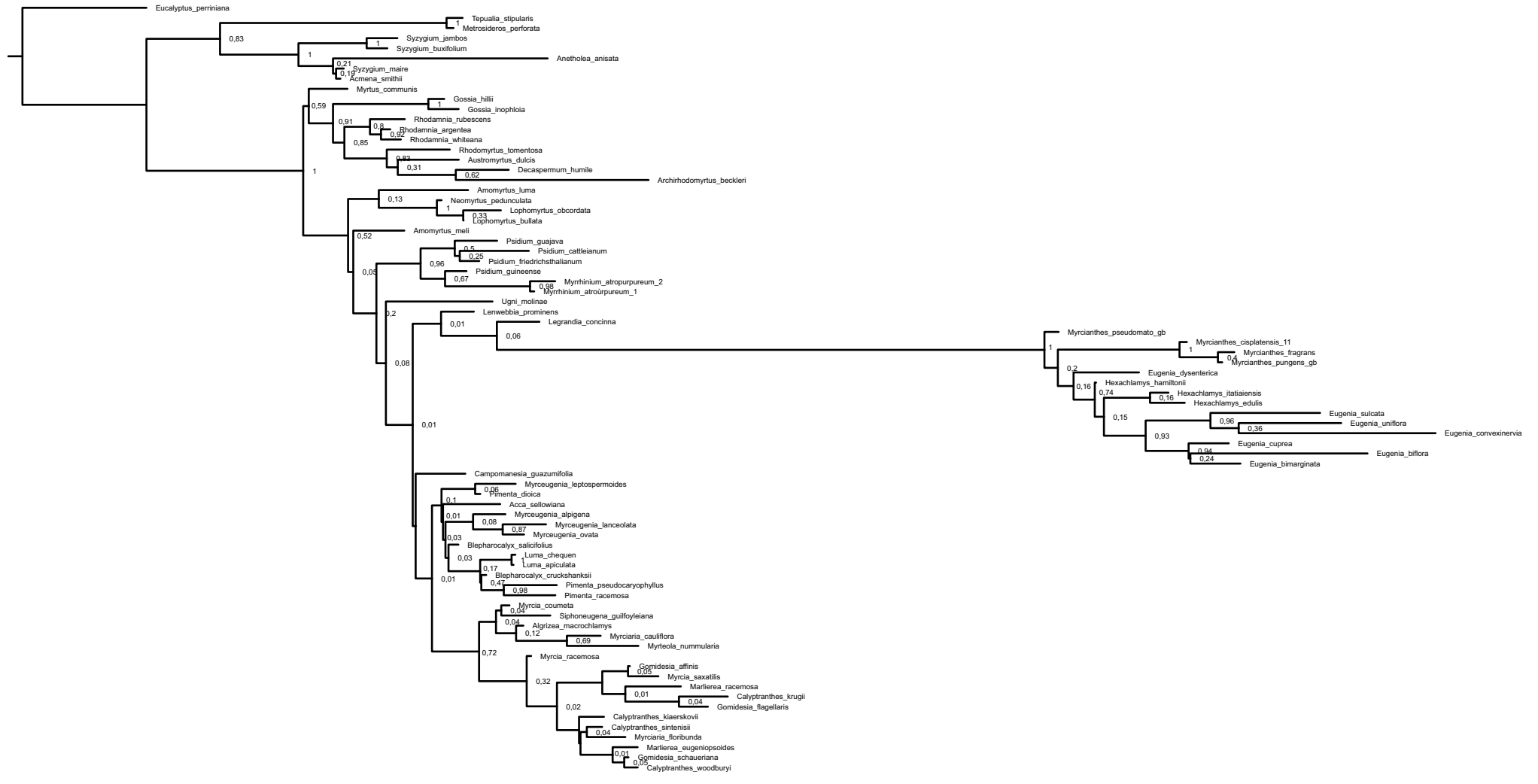

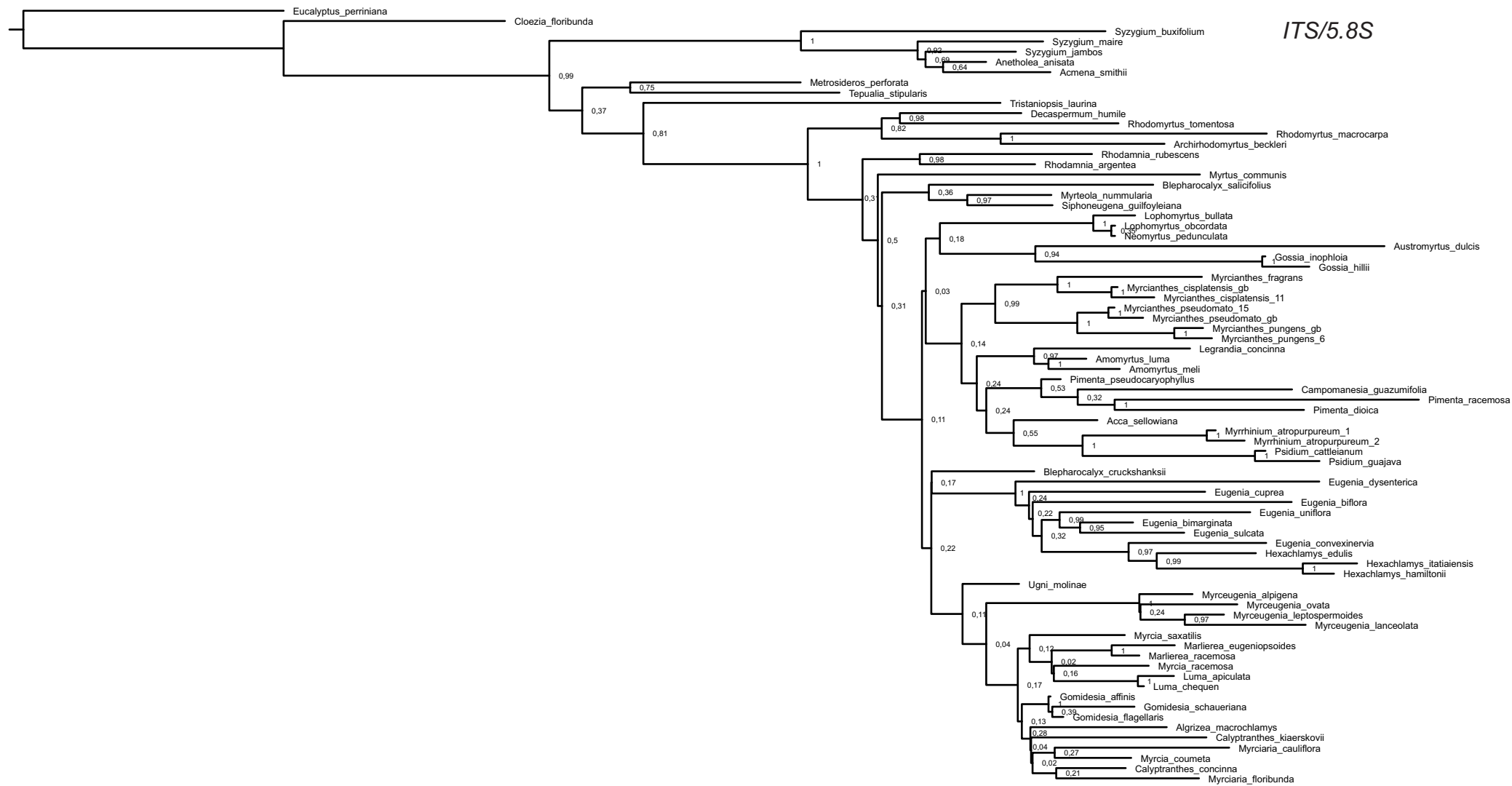

ITS/5.8S

0.08

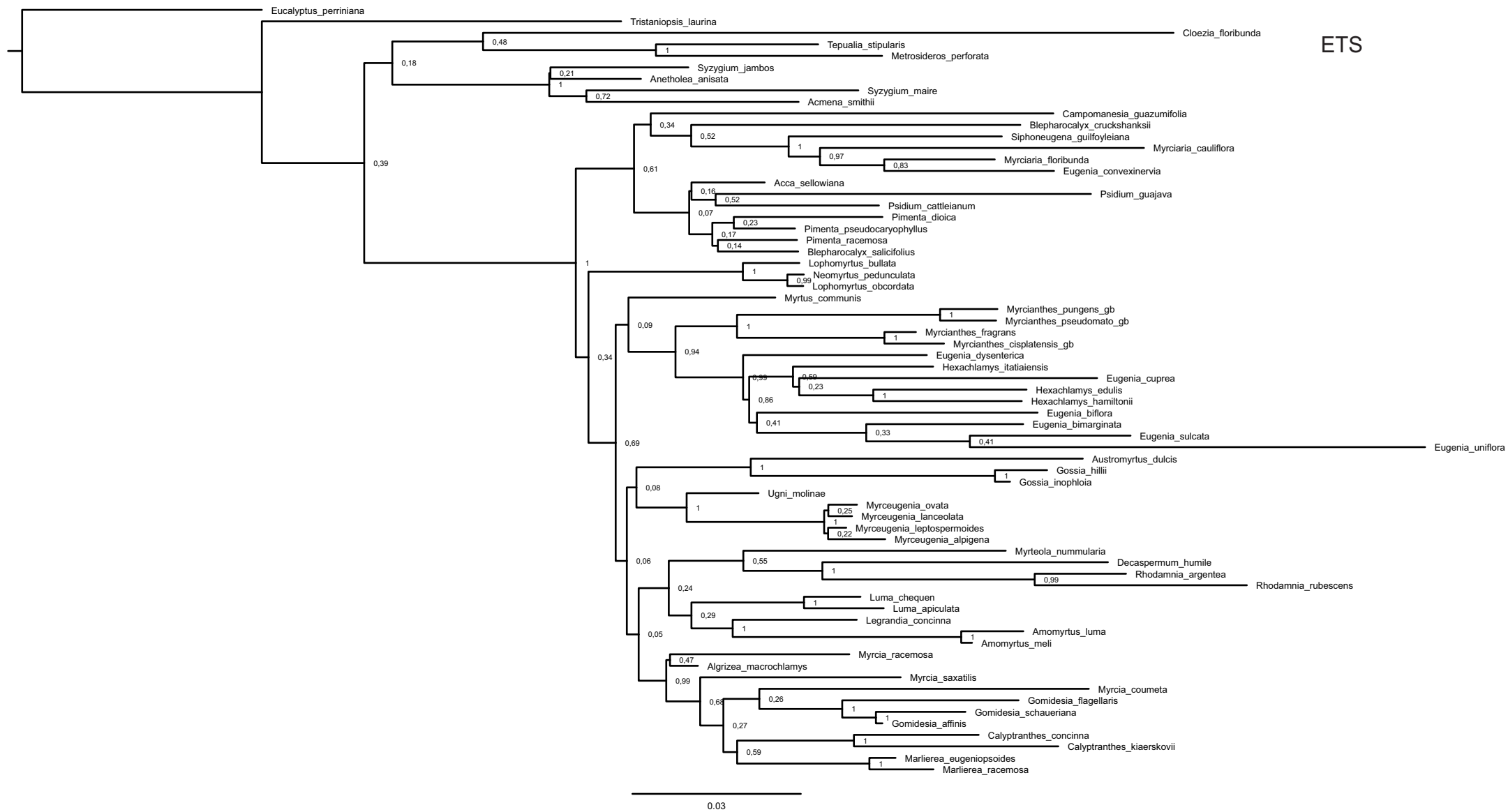

ETS

Supplement: Figure S1 — Individual analyses of the four DNA datasets (matK, psb A-trnH, ITS/5.8S and ETS). [file peerj-06-5426-s004.pdf]

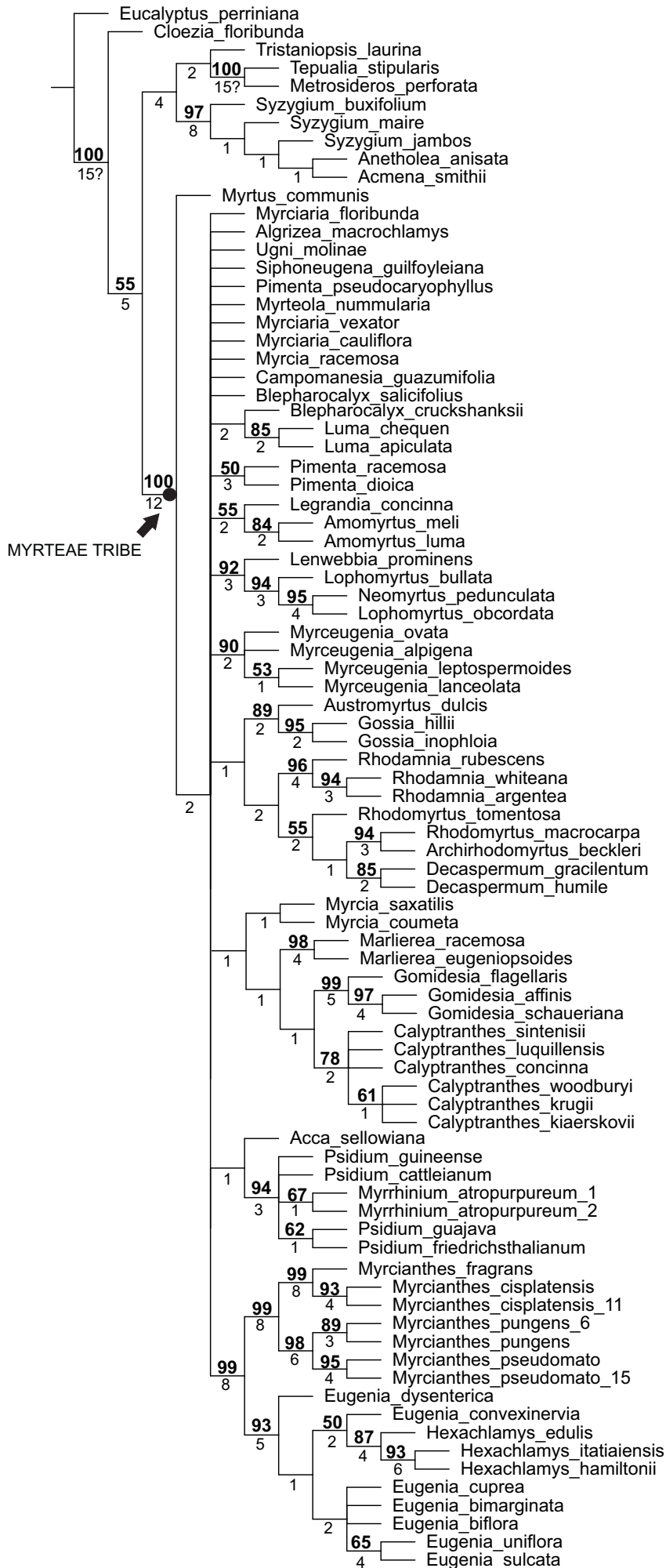

Supplement: Figure S2 — Strict consensus of 2,380 trees resulting from parsimony analysis (2,707 steps) of the combined dataset (matK, psbA-trnH, ITS/5.8S and ETS). Values on branches correspond to jackknife values above 50% (above the branch) and Bremer support values (below the branch). [file peerj-06-5426-s005.pdf]

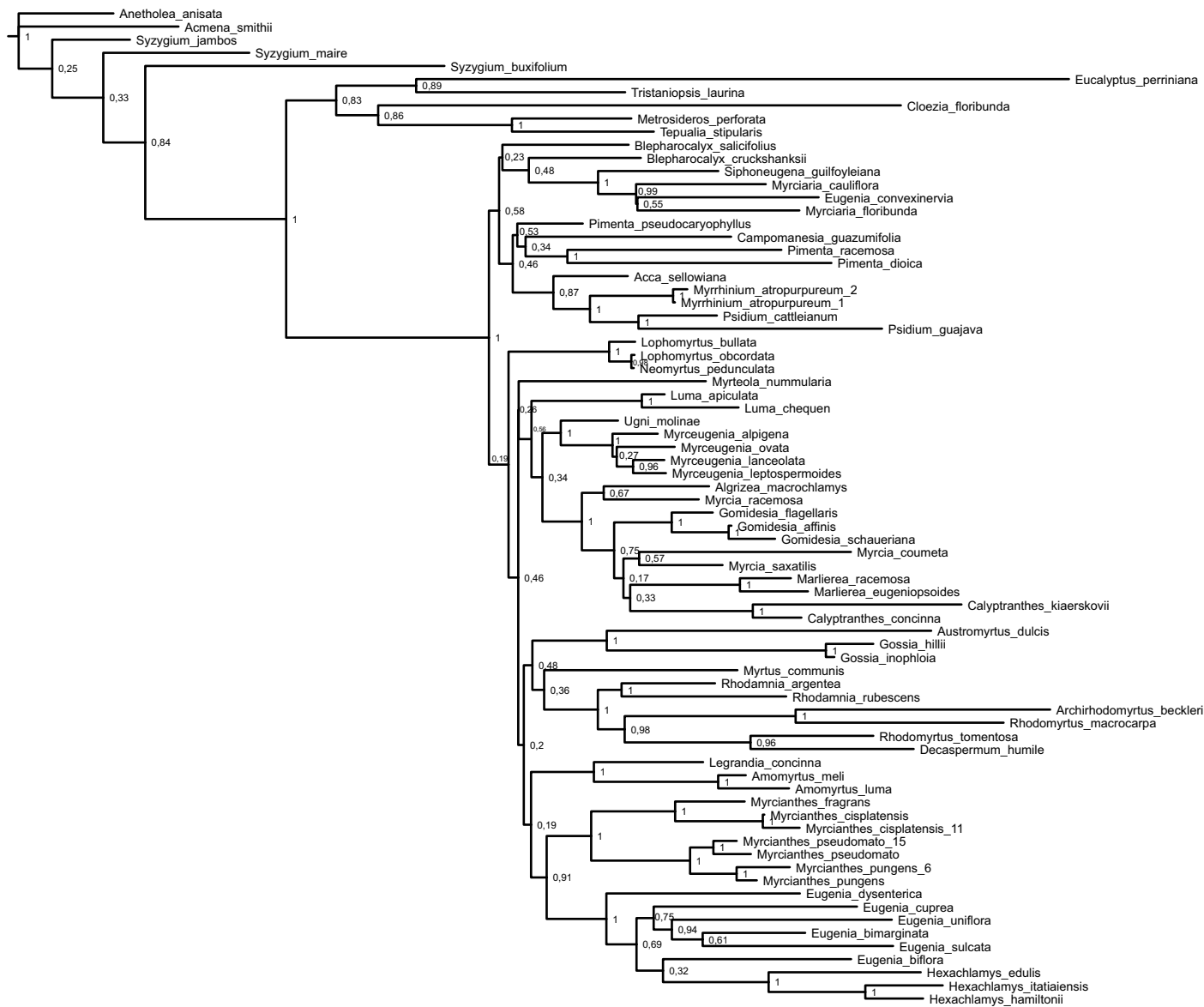

0.02

Supplement: Figure S4 — Maximum Clade Credibility Tree (from 15,002 trees) of the nuclear dataset (ITS/5.8S and ETS) obtained by Bayesian Inference. Values on nodes correspond to Bayesian posterior probability (PP). [file peerj-06-5426-s007.pdf]

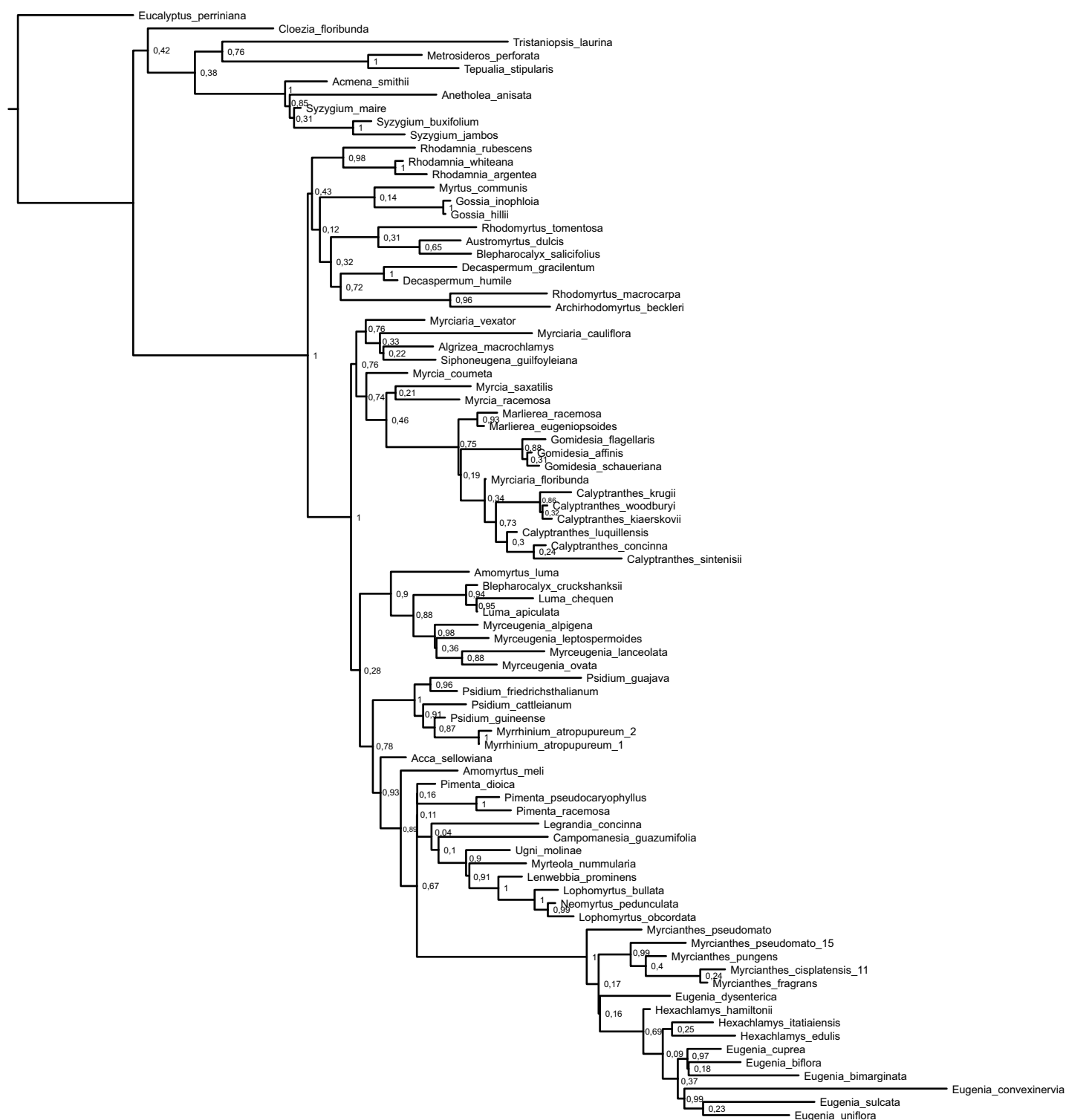

0.008

Supplement: Figure S5 — Maximum Clade Credibility Tree (from 15,002 trees) of the plastid dataset (matK and psbA-tnr H) obtained by Bayesian Inference. Values on nodes correspond to Bayesian posterior probability (PP). [file peerj-06-5426-s008.pdf]
