# Supplementary material for: Evolution of pollination by frugivorous birds in Neotropical Myrtaceae
Source: PeerJ. 2018 Aug 27;6:e5426. doi: 10.7717/peerj.5426 (PMC6118208; doi:10.7717/peerj.5426)
Supplement: Figure S3 — Full definition of characters, and details of character states see in Appendix S1. Character 0: branching degree of the paracladium; character 1: development of apical meristem of paracladium; character 2: number of first order branching within the paracladium; character 3: complexity of the first order branches within the paracladium; character 4: elongation of internodes of the floriferous branches; character 5: type of foliage supporting the paracladio; character 6: fleshy petal presence; character 7: numbers of petals; character 8: length of petals; character 9: presence of pigments in petals; character 10: number of stamens; character 11: length of stamens ; character 12: presence of purpureous pigments in filaments; character 13: main pollination type. [file peerj-06-5426-s006.pdf]

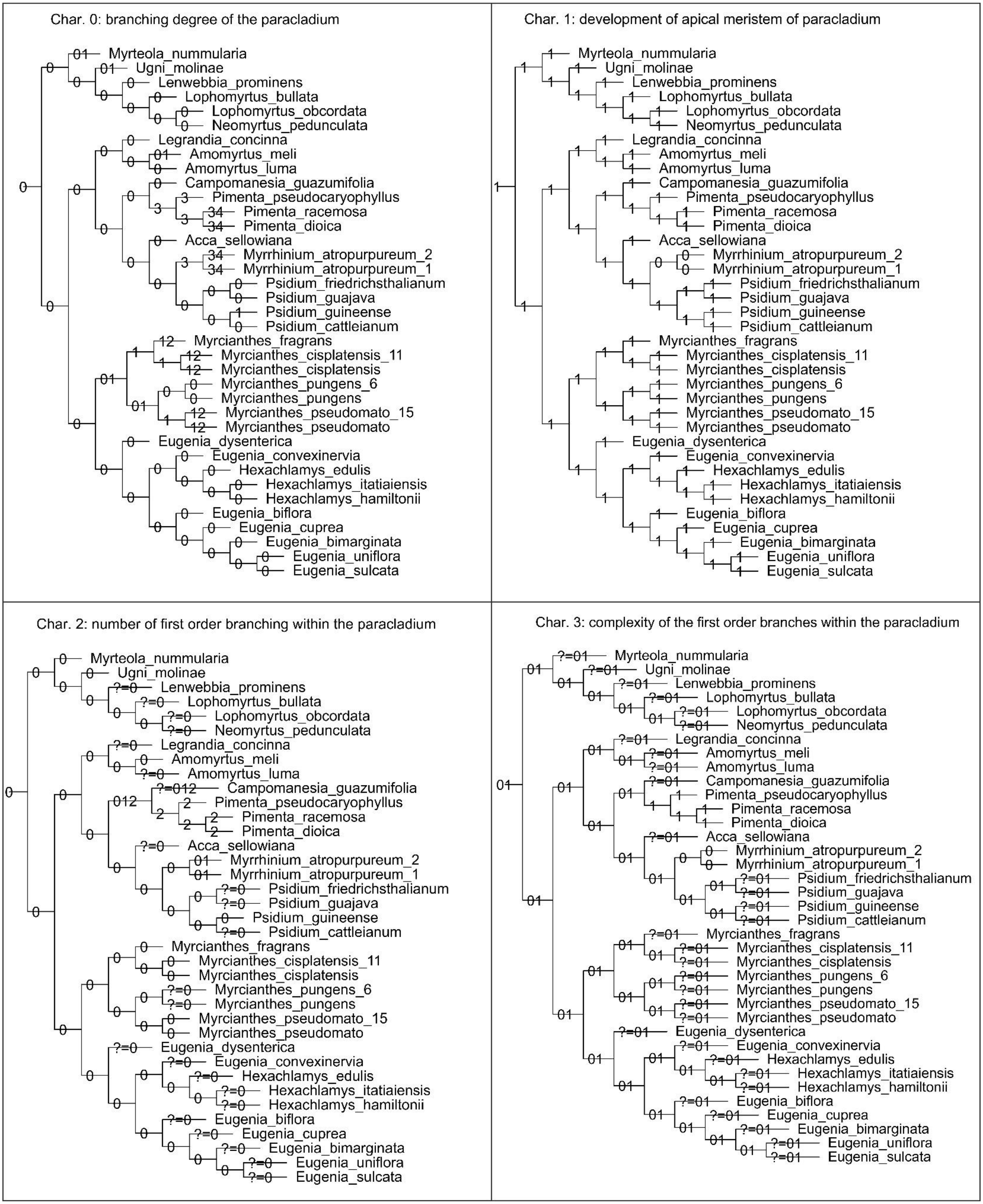
Char. 0: branching degree of the paracladium
Myrteola_nummularia
Ugni_molinae
Lenwebbia_prominens
Lophomyrtus_bullata
Lophomyrtus_obcordata
Neomyrtus_pedunculata
Legrandia_concinna
Amomyrtus_meli
Amomyrtus_luma
Campomanesia_guazumifolia
Pimenta_pseudocaryophyllus
Pimenta_racemosa
Pimenta_dioica
Acca_sellowiana
Myrrhinium_atropurpureum_2
Myrrhinium_atropurpureum_1
Psidium_friedrichsthalianum
Psidium_guajava
Psidium_guineense
Psidium_cattleianum
Myrcianthes_fragrans
Myrcianthes_cisplatensis_11
Myrcianthes_cisplatensis
Myrcianthes_pungens_6
Myrcianthes_pungens
Myrcianthes_pseudomato_15
Myrcianthes_pseudomato
Eugenia_dysenterica
Eugenia_convexinervia
Hexachlamys_edulis
Hexachlamys_itatiaiensis
Hexachlamys_hamiltonii
Eugenia_biflora
Eugenia_cuprea
Eugenia_bimarginata
Eugenia_uniflora
Eugenia_sulcata
Char. 1: development of apical meristem of paracladium
Char. 2: number of first order branching within the paracladium
Char. 3: complexity of the first order branches within the paracladium

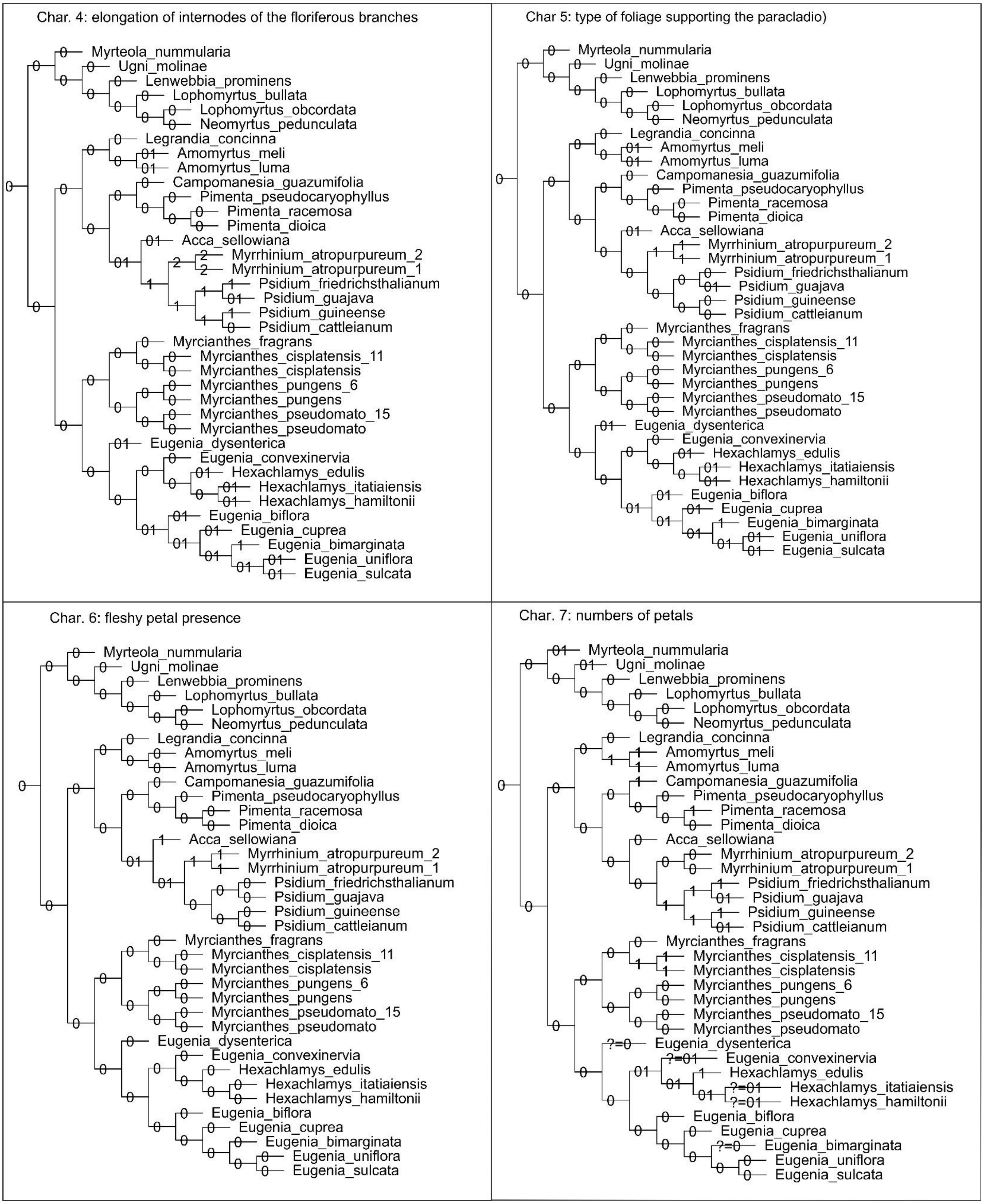
Char. 4: elongation of internodes of the floriferous branches
Myrteola_nummularia
Ugni_molinae
Lenwebbia_prominens
Lophomyrtus_bullata
Lophomyrtus_obcordata
Neomyrtus_pedunculata
Legrandia_concinna
Amomyrtus_meli
Amomyrtus_luma
Campomanesia_guazumifolia
Pimenta_pseudocaryophyllus
Pimenta_racemosa
Pimenta_dioica
Acca_sellowiana
Myrrhinium_atropurpureum_2
Myrrhinium_atropurpureum_1
Psidium_friedrichsthalianum
Psidium_guajava
Psidium_guineense
Psidium_cattleianum
Myrcianthes_fragrans
Myrcianthes_cisplatensis_11
Myrcianthes_cisplatensis
Myrcianthes_pungens_6
Myrcianthes_pungens
Myrcianthes_pseudomato_15
Myrcianthes_pseudomato
Eugenia_dysenterica
Eugenia_convexinervia
Hexachlamys_edulis
Hexachlamys_itatiaiensis
Hexachlamys_hamiltonii
Eugenia_biflora
Eugenia_cuprea
Eugenia_bimarginata
Eugenia_uniflora
Eugenia_sulcata
Char 5: type of foliage supporting the paracladio)
Myrteola_nummularia
Ugni_molinae
Lenwebbia_prominens
Lophomyrtus_bullata
Lophomyrtus_obcordata
Neomyrtus_pedunculata
Legrandia_concinna
Amomyrtus_meli
Amomyrtus_luma
Campomanesia_guazumifolia
Pimenta_pseudocaryophyllus
Pimenta_racemosa
Pimenta_dioica
Acca_sellowiana
Myrrhinium_atropurpureum_2
Myrrhinium_atropurpureum_1
Psidium_friedrichsthalianum
Psidium_guajava
Psidium_guineense
Psidium_cattleianum
Myrcianthes_fragrans
Myrcianthes_cisplatensis_11
Myrcianthes_cisplatensis
Myrcianthes_pungens_6
Myrcianthes_pungens
Myrcianthes_pseudomato_15
Myrcianthes_pseudomato
Eugenia_dysenterica
Eugenia_convexinervia
Hexachlamys_edulis
Hexachlamys_itatiaiensis
Hexachlamys_hamiltonii
Eugenia_biflora
Eugenia_cuprea
Eugenia_bimarginata
Eugenia_uniflora
Eugenia_sulcata
Char. 6: fleshy petal presence
Myrteola_nummularia
Ugni_molinae
Lenwebbia_prominens
Lophomyrtus_bullata
Lophomyrtus_obcordata
Neomyrtus_pedunculata
Legrandia_concinna
Amomyrtus_meli
Amomyrtus_luma
Campomanesia_guazumifolia
Pimenta_pseudocaryophyllus
Pimenta_racemosa
Pimenta_dioica
Acca_sellowiana
Myrrhinium_atropurpureum_2
Myrrhinium_atropurpureum_1
Psidium_friedrichsthalianum
Psidium_guajava
Psidium_guineense
Psidium_cattleianum
Myrcianthes_fragrans
Myrcianthes_cisplatensis_11
Myrcianthes_cisplatensis
Myrcianthes_pungens_6
Myrcianthes_pungens
Myrcianthes_pseudomato_15
Myrcianthes_pseudomato
Eugenia_dysenterica
Eugenia_convexinervia
Hexachlamys_edulis
Hexachlamys_itatiaiensis
Hexachlamys_hamiltonii
Eugenia_biflora
Eugenia_cuprea
Eugenia_bimarginata
Eugenia_uniflora
Eugenia_sulcata
Char. 7: numbers of petals
Myrteola_nummularia
Ugni_molinae
Lenwebbia_prominens
Lophomyrtus_bullata
Lophomyrtus_obcordata
Neomyrtus_pedunculata
Legrandia_concinna
Amomyrtus_meli
Amomyrtus_luma
Campomanesia_guazumifolia
Pimenta_pseudocaryophyllus
Pimenta_racemosa
Pimenta_dioica
Acca_sellowiana
Myrrhinium_atropurpureum_2
Myrrhinium_atropurpureum_1
Psidium_friedrichsthalianum
Psidium_guajava
Psidium_guineense
Psidium_cattleianum
Myrcianthes_fragrans
Myrcianthes_cisplatensis_11
Myrcianthes_cisplatensis
Myrcianthes_pungens_6
Myrcianthes_pungens
Myrcianthes_pseudomato_15
Myrcianthes_pseudomato
Eugenia_dysenterica
Eugenia_convexinervia
Hexachlamys_edulis
Hexachlamys_itatiaiensis
Hexachlamys_hamiltonii
Eugenia_biflora
Eugenia_cuprea
Eugenia_bimarginata
Eugenia_uniflora
Eugenia_sulcata

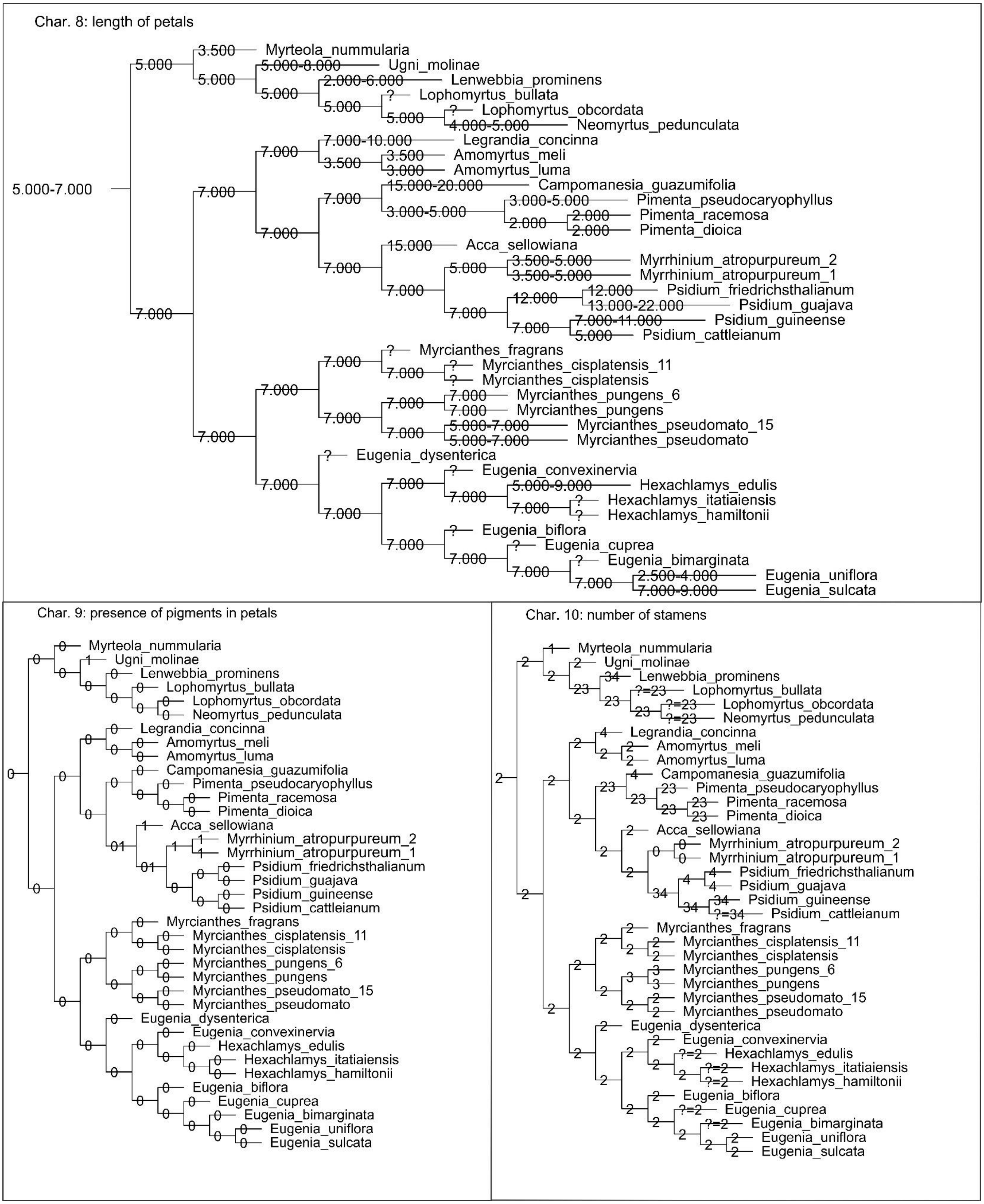
Char. 8: length of petals
Myrteola_nummularia
Ugni_molinae
Lenwebbia_prominens
Lophomyrtus_bullata
Lophomyrtus_obcordata
Neomyrtus_pedunculata
Legrandia_concinna
Amomyrtus_meli
Amomyrtus_luma
Campomanesia_guazumifolia
Pimenta_pseudocaryophyllus
Pimenta_racemosa
Pimenta_dioica
Acca_sellowiana
Myrrhinium_atropurpureum_2
Myrrhinium_atropurpureum_1
Psidium_friedrichsthalianum
Psidium_guajava
Psidium_guineense
Psidium_cattleianum
Myrcianthes_fragrans
Myrcianthes_cisplatensis_11
Myrcianthes_cisplatensis
Myrcianthes_pungens_6
Myrcianthes_pungens
Myrcianthes_pseudomato_15
Myrcianthes_pseudomato
Eugenia_dysenterica
Eugenia_convexinervia
Hexachlamys_edulis
Hexachlamys_itatiaiensis
Hexachlamys_hamiltonii
Eugenia_biflora
Eugenia_cuprea
Eugenia_bimarginata
Eugenia_uniflora
Eugenia_sulcata
Char. 9: presence of pigments in petals
Char. 10: number of stamens

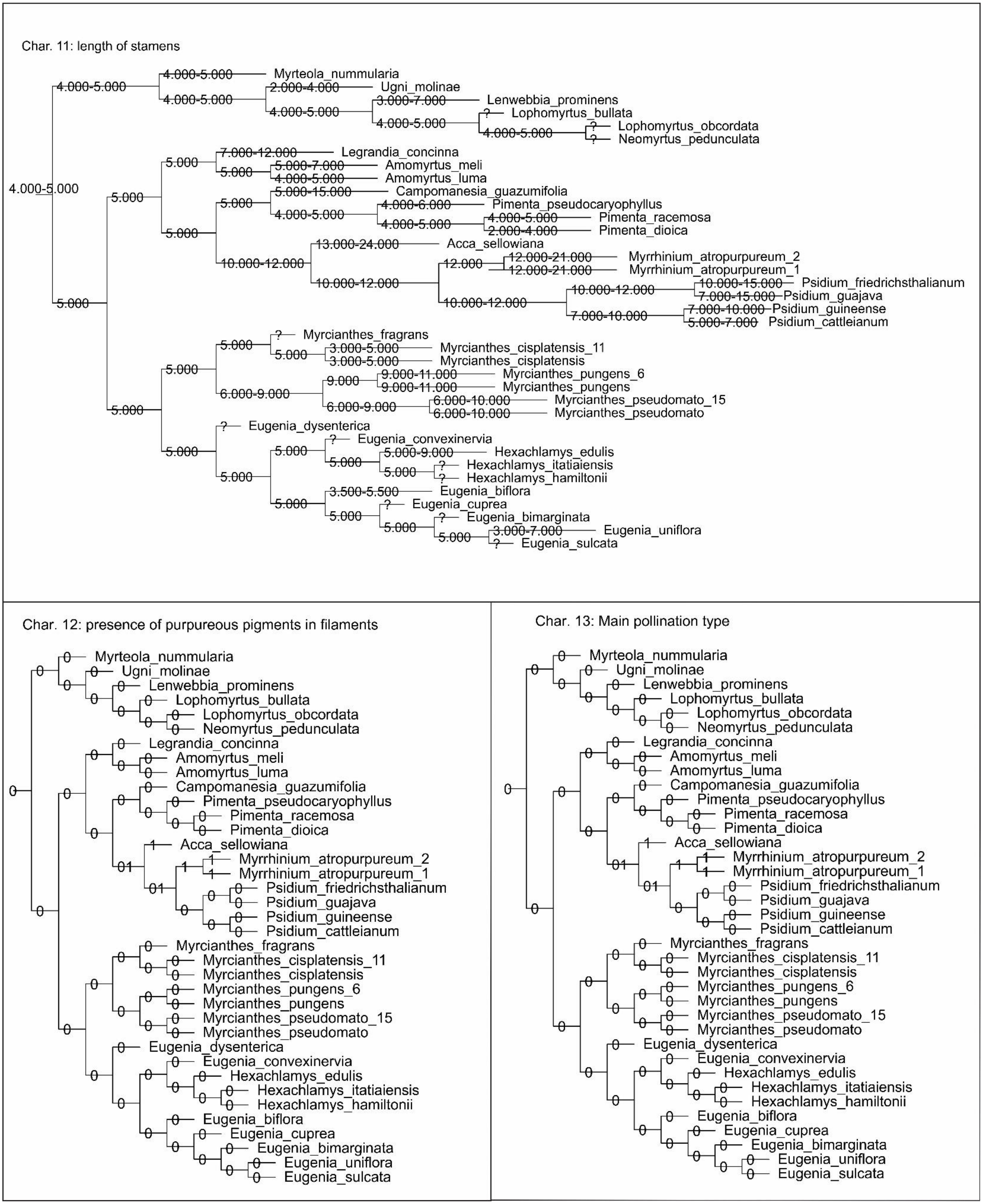
Char. 11: length of stamens
Myrteola_nummularia
Ugni_molinae
Lenwebbia_prominens
Lophomyrtus_bullata
Lophomyrtus_obcordata
Neomyrtus_pedunculata
Legrandia_concinna
Amomyrtus_meli
Amomyrtus_luma
Campomanesia_guazumifolia
Pimenta_pseudocaryophyllus
Pimenta_racemosa
Pimenta_dioica
Acca_sellowiana
Myrrhinium_atropurpureum_2
Myrrhinium_atropurpureum_1
Psidium_friedrichsthalianum
Psidium_guajava
Psidium_guineense
Psidium_cattleianum
Myrcianthes_fragrans
Myrcianthes_cisplatensis_11
Myrcianthes_cisplatensis
Myrcianthes_pungens_6
Myrcianthes_pungens
Myrcianthes_pseudomato_15
Myrcianthes_pseudomato
Eugenia_dysenterica
Eugenia_convexinervia
Hexachlamys_edulis
Hexachlamys_itatiaiensis
Hexachlamys_hamiltonii
Eugenia_biflora
Eugenia_cuprea
Eugenia_bimarginata
Eugenia_uniflora
Eugenia_sulcata
Char. 12: presence of purpureous pigments in filaments
Char. 13: Main pollination type
